# Addition of four new wood-inhabiting fungi of *Russulales* (*Agaricomycetes*, *Basidiomycota*) from southwestern China

**DOI:** 10.3897/mycokeys.133.193737

**Published:** 2026-05-27

**Authors:** Yinglian Deng, Kaisheng Wang, Meng Chen, Yuhan Qiu, Wanting Liu, Sana Jabeen, Xiangfu Liu, Changlin Zhao

**Affiliations:** 1 Key Laboratory of Forest Disaster Warning and Control in Universities of Yunnan Province, Southwest Forestry University, Kunming 650224, China Department Microbial Drugs (MWIS), Helmholtz-Centre for Infection Research Braunschweig Germany; 2 College of Forestry, Southwest Forestry University, Kunming 650224, China College of Forestry, Southwest Forestry University Kunming China; 3 Department of Botany, Division of Science and Technology, University of Education, Township, Lahore, Punjab, Pakistan Key Laboratory of Forest Disaster Warning and Control in Universities of Yunnan Province, Southwest Forestry University Kunming China; 4 Modern Industry School of Edible-fungi, Southwest Forestry University, Kunming 650224, China Modern Industry School of Edible-fungi, Southwest Forestry University Kunming China; 5 Department Microbial Drugs (MWIS), Helmholtz-Centre for Infection Research, 38124 Braunschweig, Germany Department of Botany, Division of Science and Technology, University of Education Lahore Pakistan

**Keywords:** Biodiversity, coriaceous fungi, molecular systematics, *
Peniophoraceae
*, *
Stereaceae
*, taxonomy

## Abstract

The species diversity of the families *Peniophoraceae* and *Stereaceae (Russulales)* in the high-altitude forests of southwestern China remains poorly explored. In this study, the detailed morphological characteristics and combined two-locus phylogenetic analyses based on the internal transcribed spacer (ITS) and large subunit (nLSU) regions of nuclear ribosomal DNA (rDNA) revealed four new wood-inhabiting species from southwestern China, belonging to the genera *Baltazaria*, *Scytinostroma*, *Vararia*, and *Xylobolus*. Their morphology and combined ITS + nLSU dataset showed that three of them grouped within *Peniophoraceae*, viz., *Baltazaria yunnanensis*, *Scytinostroma sinense*, and *Vararia dehongensis*, whereas *Xylobolus yunnanensis* clustered within *Stereaceae*. Descriptions, illustrations, and phylogenetic analysis results of the new taxa are provided. This study expands knowledge of *Russulales* diversity in China and provides baseline data for the conservation and sustainable utilization of fungal resources.

## Introduction

The systematics of fungi has been revolutionized by advancements in molecular biology, phylogenetics, and bioinformatics (Nagy et al. 2025; Wiens et al. 2026). Currently, morphology integrated with DNA sequence-based classification and identification has become the standard approach in fungal taxonomy (Yurchenko et al. 2020; Hyde et al. 2024; Zou et al. 2025). Approximately 155,000 fungal species have been described, and possibly millions more remain unnamed (Hibbett et al. 2025). The monophyly of *Russulales* Kreisel ex P.M. Kirk, P.F. Cannon & J.C. David, with the type family *Russulaceae* Lotsy, has been supported by morphological and multi-locus DNA phylogenetic analyses (Hibbett et al. 1997; Kirk et al. 2001; Binder et al. 2005; Miller et al. 2006; Wang et al. 2025), and consequently, an increasing number of species within the order have been described through integrative taxonomic approaches combining morphology and DNA-based phylogenetics (Zhou and Dai 2013; Chen et al. 2015, 2016a, 2016b; Wu et al. 2020; Yuan et al. 2021; Dong et al. 2025a, 2025b). It is a highly diverse group of *Basidiomycota*, and approximately 4500 species have been ascribed to the order, representing 98 genera within 11 families (He et al. 2024; Bao et al. 2025). Members of *Russulales* exhibit a wide range of ecological strategies, including saprotrophic wood decay, tree root and heartwood pathogens, ectomycorrhizal symbiosis, and association with insects (Hibbett et al. 2014; Dong et al. 2024). Among these taxa, resupinate basidiomata are frequently encountered in several families, particularly within *Peniophoraceae* Lotsy and *Stereaceae* Pilát (Wang et al. 2025, 2026; Deng et al. 2026).

*Peniophoraceae* was established by Lotsy (1907) and typified by *Peniophora* Cooke. Analyses based on ITS1-5.8S-ITS2-nLSU nuclear rDNA revealed that the taxa of *Peniophoraceae* were nested in the russuloid clade, which holds a considerable share of the phylogenetic framework (Larsson and Larsson 2003; Larsson et al. 2004; Larsson 2007; Leal-Dutra et al. 2018; Nagy et al. 2025). According to recent research on molecular systematics, *Peniophoraceae* includes 17 genera, but only a few genera form distinct monophyletic lines (Dai 2011; He et al. 2024; Deng et al. 2024a, 2024b). Within *Peniophoraceae*, *Peniophora*, *Scytinostroma* Donk, and *Vararia* P. Karst. formed the main clades; moreover, morphologically, *Scytinostroma* was similar to *Vararia*, which usually differed in having typical dichohyphae (Bernicchia and Gorjón 2010). The taxonomic distinction between *Scytinostroma* and *Vararia* has been questioned (Hallenberg and Eriksson 1985; Boidin and Lanquetin 1987; Stalpers 1996; Boidin et al. 1998; Kirk et al. 2008; Tedersoo et al. 2014; Deng et al. 2024b). However, there has been general agreement that the two genera were closely related and that they together made up a natural group. Larsson and Larsson (2003) strongly suggested that neither skeletal hyphae nor their branching patterns have any predictive power in a phylogenetic context. Therefore, more *Scytinostroma* and *Vararia* species need to be introduced to more clearly define the relationships among them.

*Vararia* is a widely distributed corticioid genus typified by *V. investiens* (Karnste 1898; Boidin and Lanquetin 1975; Karasinski 2010; Yurchenko et al. 2020). In recent years, based on a combination of morphoanatomical characteristics and molecular phylogenetic data, new *Vararia* species have been constantly reported, occurring mainly in tropical and subtropical areas of the world (Bernicchia and Gorjón 2010; Nakasone 2015; Liu and He 2016; Dai et al. 2021; Deng et al. 2024b; Wang et al. 2026). Multi-gene phylogenetic analyses revealed that *Vararia* is paraphyletic, but available morphological evidence remains insufficient to justify splitting the genus into separate genera (Zou et al. 2022; Deng et al. 2024b). Accordingly, the discovery and formal description of additional species are needed to better resolve species boundaries and evolutionary relationships within *Vararia*.

Morphologically, species of *Baltazaria* Leal-Dutra, Dentinger & G.W. Griff. and *Scytinostroma* have particularly similar morphological characteristics, but molecular evidence demonstrated that they formed completely different branches (Leal-Dutra et al. 2018). *Baltazaria* is characterized by corticioid, adherent to effused, coriaceous, or membranaceous to hard basidiomata and a white, cream, or pale ochraceous hymenial surface. The context is densely homogeneous with thick-walled and dextrinoid skeletal-binding hyphae, sometimes bearing rows of short papillae or skeletodendrohyphidia (Leal-Dutra et al. 2018). Currently, only five *Baltazaria* species, *B. octopodites* (Corner) Leal-Dutra, Dentinger & G.W. Griff., *B. galactina*, *B. neogalactina* (Boidin & Lanq.) Leal-Dutra, Dentinger & G.W. Griff., *B. eurasiaticogalactina*, and *B. pingbianensis* Y.L.Deng & C.L. Zhao, have been reported worldwide based on morphological characteristics and phylogenetic analyses (Leal-Dutra et al. 2018). In contrast, species in the genus *Scytinostroma* have a dimitic hyphal system with simple septa or clamps on generative hyphae, skeletal hyphae densely branched and sometimes forming dendrohyphae or dichohyphae, and subglobose to ellipsoid basidiospores (Donk 1956; Bernicchia and Gorjón 2010; Wang et al. 2020; Zhang et al. 2023; Ji et al. 2024). Based on concatenated ITS1-5.8S-ITS2-nLSU sequence data, with a focus on samples of *Scytinostroma* s.s. in different localities, an increasing number of new *Scytinostroma* species has been reported (Li et al. 2023; Dong et al. 2024). Currently, 63 specific and infraspecific names are recorded in Index Fungorum (accessed 1 May 2026), of which 44 are accepted worldwide (Zhang et al. 2023; Li et al. 2023; Dong et al. 2024; Ji et al. 2024).

*Stereaceae*, typified by *Stereum* Hill ex Pers., formed a well-supported monophyletic clade in *Russulales* (Larsson and Larsson 2003; Miller et al. 2006; Larsson 2007), with 23 genera currently accepted (Xu et al. 2025). Species of *Stereaceae* are all saprobic and cause white rot on both conifers and hardwoods (He et al. 2024). *Xylobolus* P. Karst. is a small, globally distributed wood-inhabiting genus classified within the family *Stereaceae (Russulales)* (Cao and He 2020). The type species of the genus is *X. frustulatus* (Pers.) P. Karst., a distinctive corticioid species that grows on dead or decaying oak wood and forms perennial basidiomata, which crack into small, angular polygons (Felegyi et al. 2023). There are few taxonomic studies on *Xylobolus*, although the chemical composition of *Xylobolus* has been studied (Felegyi et al. 2023). Currently, 18 specific and infraspecific names are recorded in Index Fungorum (accessed 1 May 2026), of which 10 are accepted worldwide (Berkeley and Curtis 1849; Felegyi et al. 2023; Xu et al. 2025).

During investigations of wood-inhabiting fungi in Yunnan, China, specimens representing four new species belonging to *Baltazaria*, *Scytinostroma*, *Vararia*, and *Xylobolus* were collected. To clarify their phylogenetic placement and relationships, phylogenetic and taxonomic analyses of the four species were conducted based on ITS and nLSU sequences.

## Materials and methods

### Sample collection and herbarium specimen preparation

Fresh basidiomata were collected from Dehong, Pu’er, Wenshan, and Tengchong in Yunnan, China. The samples were photographed in situ, and fresh macroscopic details were recorded. Photographs were taken with a Nikon D7100 camera. All photographs were focus-stacked using Helicon Focus software. Macroscopic details were recorded, and the samples were transported to a field station where the fruit bodies were dried in an electronic food dryer at 45 °C (Dong et al. 2024). Once dried, the specimens were sealed in envelopes and zip-lock plastic bags and labeled (Yang et al. 2025). The dried specimens were deposited in the Herbarium of Southwest Forestry University (SWFC), Kunming, Yunnan Province, China.

### Morphology

Macro-morphological descriptions were based on field notes and photographs captured in the field and the laboratory. Color terminology followed Petersen (1996). Micro-morphological data were obtained from dried specimens observed using a light microscope at 1000× magnification with oil immersion (Zhao et al. 2023; Dong et al. 2024). The following abbreviations are used: **KOH** = 5% potassium hydroxide aqueous solution, **CB** = cotton blue, **CB–** = acyanophilous, **IKI** = Melzer’s reagent, **IKI+** = amyloid, **IKI–** = both inamyloid and indextrinoid, and **SA–** = negative reaction in sulfobenzaldehyde. ***L*** = mean spore length (arithmetic average for all spores), ***W*** = mean spore width (arithmetic average for all spores), ***Q*** = variation in the *L*/*W* ratios between the specimens studied, and ***n*** = *a*/*b* (number of spores (*a*) measured from a given number (*b*) of specimens). A minimum of 30 basidiospores from each specimen was measured. Stalks were excluded from basidia measurements, and the hilar appendage was excluded from basidiospore measurements. The MycoBank numbers were registered in the MycoBank database (http://www.mycobank.org).

### Molecular phylogeny

The EZNA HP Fungal DNA Kit (Omega Biotechnologies Co., Ltd., Kunming, China) was used to extract DNA, with some modifications, from the dried specimens. The nuclear ribosomal ITS region was amplified with primers ITS5 and ITS4 (White et al. 1990). The PCR procedure for ITS was as follows: initial denaturation at 95 °C for 3 min, followed by 35 cycles at 94 °C for 40 s, 58 °C for 45 s, and 72 °C for 1 min, and a final extension at 72 °C for 10 min. The nuclear LSU region was amplified with the primer pair LR0R and LR7 (Vilgalys and Hester 1990; Rehner and Samuels 1994). The PCR procedure for LSU was as follows: initial denaturation at 94 °C for 1 min, followed by 35 cycles at 94 °C for 30 s, 48 °C for 1 min, and 72 °C for 1.5 min, and a final extension at 72 °C for 10 min. The PCR procedure for ITS and LSU followed a previous study (Zhao and Wu 2017). All newly generated sequences were deposited in NCBI GenBank (https://www.ncbi.nlm.nih.gov/genbank/) (Table 1).

**Table 1.** List of species, specimens, and GenBank accession numbers of sequences used in this study. “*” indicates type materials, and “—” indicates data unavailability.

Species name	Sample no.	GenBank Accession No.		Country	References
		ITS	nLSU		
*Acanthobasidium bambusicola*	He2357	KU559343	KU574833	China	Dai and He (2016)
*Acanthobasidium delicatum*	CBS 233.86	—	MH873638	France	Vu et al. (2019)
*Acanthobasidium norvegicum*	T623	—	AY039328	France	Wu et al. (2001)
*Acanthobasidium quilae*	SPG3088	MT831059	—	Chile	Rajchenberg et al. (2021)
*Acanthobasidium weirii*	HHB13132	KX306882	—	Italy	Dai et al. (2017a)
*Acanthobasidium weirii*	HHB13499	KX306883	—	Canada	Dai et al. (2017a)
*Acanthofungus rimosus*	Wu9601-1	MF043521	AY039333	China	Wu et al. (2019)
*Aleurobotrys botryosus*	DAOM211598	AF506398	AF506398	Canada	Larsson and Larsson (2003)
*Aleurobotrys botryosus*	He2712	KX306877	KY450788	China	Dai et al. (2017b)
*Aleurodiscus amorphus*	He4937	MW533080	MW528916	China	Xu et al. (2025)
*Aleurodiscus amorphus*	HHB15282	—	AY039312	USA	Wu et al. (2001)
*Aleurodiscus gigasporus*	He2865	MW533081	MW528917	China	Xu et al. (2025)
*Aleurodiscus gigasporus*	Wu0108-15	KY706205	KY706213	China	Dai and He (2017)
*Aleurodiscus grntii*	He2895	KU559347	KU574837	China	Dai and He (2016)
*Aleurodiscus grntii*	T569	—	AY039326	USA	Wu et al. (2001)
*Aleurodiscus penicilltus*	T322	—	AY039315	Canada	Wu et al. (2001)
*Aleurodiscus pinicola*	Wu1106-16	MF043524	MF043529	China	Wu et al. (2019)
*Aleurodiscus pinicola*	Wu1308-54	MF043525	MF043530	China	Wu et al. (2019)
*Aleurodiscus subroseus*	He4807	MH109054	MH109048	China	Tian et al. (2018)
*Aleurodiscus wakefieldiae*	He2579	KU559355	KU574843	China	Dai and He (2016)
*Baltazaria galactina*	CBS 752.86	MH862034	MH873721	France	Vu et al. (2019)
*Baltazaria galactina*	He4999	MK625618	MK625547	France	Vu et al. (2019)
*Baltazaria neogalactina*	CBS 755.86	MH862037	MH873724	France	Vu et al. (2019)
*Baltazaria neogalactina*	CBS:758.86	MH862040	MH873727	France	Vu et al. (2019)
*Baltazaria octopodites*	FLOR56460	MH260032	MH260050	UK	Leal-Dutra et al. (2018)
*Baltazaria octopodites*	INPA280140	MH260038	MH260056	UK	Leal-Dutra et al. (2018)
*Baltazaria pingbianensis*	CLZhao 17755*	OR048814	OR510674	China	Deng et al. (2026)
*Baltazaria pingbianensis*	CLZhao 18294	OR048816	—	China	Deng et al. (2026)
*Baltazaria pingbianensis*	CLZhao 18296	OR048815	—	China	Deng et al. (2026)
***Baltazaria yunnanensis***	CLZhao 17182*	**PZ013432**	—	**China**	**Present study**
*Conferticium ochraceum*	He2900	KY860409	KY860467	China	Unpublished
*Conferticium ochraceum*	He2932	KY860411	KY860470	China	Unpublished
*Conferticium subtropicum*	He1804	KY860404	MW528922	China	Xu et al. (2025)
*Conferticium subtropicum*	He1827^*^	KY860405	KY860463	China	Unpublished
*Confertobasidium olivaceoalbum*	FP 90196	AF511648	AF511648	Sweden	Larsson and Larsson (2003)
*Confertotrama macrospora*	Wu9202-21^*^	AF506377	AF506377	China	Larsson and Larsson (2003)
*Confertotrama rajchenbergii*	NH16358	JQ716940	—	Chile	Gorjón and Hallenberg (2013)
*Confertotrama rugulosa*	He3427	MW533086	MW528925	China	Xu et al. (2025)
*Confertotrama rugulosa*	He5997	—	MW528924	China	Xu et al. (2025)
*Gelatinostereum phlebioides*	Dai14965^*^		MW528940	China	Xu et al. (2025)
*Gelatinostereum phlebioides*	He1951	MW533097	MW528943	China	Xu et al. (2025)
*Gloeocystidiopsis heimii*	CBS 321.66	AF506381	AF506381	Central African Republic	Larsson and Larsson (2003)
*Gloeocystidiopsis heimii*	He5830	MW533085	MW528921	Sri Lanka	Xu et al. (2025)
*Gloeocystidiopsis ravum*	CBS 125849	MH863805	MH875269	Estonia	Vu et al. (2019)
*Gloeocystidiopsis ravum*	NH13291	AF506382	AF506382	Estonia	Larsson and Larsson (2003)
*Gloeocystidiopsis shenghuae*	He5411^*^	MW533083	MW528919	China	Xu et al. (2025)
*Gloeocystidiopsis tenuissimus*	He3575^*^	KX306880	KY706214	China	Dai et al. (2017a)
*Gloeocystidiopsis tenuissimus*	He4816	—	MW528923	China	Xu et al. (2025)
*Gloeomyces bicornis*	Wu1308-101	LC433893	LC433900	China	Wu et al. (2022)
*Gloeomyces bicornis*	Wu1308-125^*^	LC433899	LC433906	China	Wu et al. (2022)
*Gloeomyces dextrinoideophyses*	He4078^*^	—	KY450783	Thailand	Dai et al. (2017b)
*Gloeomyces formosanus*	Chen2736^*^	LC433894	LC433901	China	Wu et al. (2022)
*Gloeomyces formosanus*	Chen2748	LC433895	LC433902	China	Wu et al. (2022)
*Gloeomyces graminicola*	He2606	KY860401	KY860459	China	Unpublished
*Gloeomyces parvisporus*	Wu1307-84	LC433897	LC433904	China	Wu et al. (2022)
*Gloeomyces parvisporus*	Wu1307-88	LC433898	LC433905	China	Wu et al. (2022)
*Gloeomyces persicus*	Ghobad-Nejhad3173^*^	KU213590	KU213591	China	Ghobad-Nejhad and Langer (2018)
*Gloeomyces subcerussatus*	Ghobad-Nejhad2360^*^	MH109051	MH109045	China	Tian et al. (2018)
*Gloeomyces subcerussatus*	He6964	MW533099	MW528948	China	Xu et al. (2025)
*Gloeomyces thailandicus*	He5307	MW533082	MW528918	China	Xu et al. (2025)
*Gloeomyces thoenii*	CBS 236.86	MH861950	MH873640	Netherlands	Vu et al. (2019)
*Gloeomyces tropicus*	He3830^*^	KX553875	KX578720	China	Dai et al. (2017a)
*Gloeosoma decorticans*	MR12665	MT831042	MT831022	Chile	Rajchenberg et al. (2021)
*Gloeosoma decorticans*	MR12666	MT831041	MT831021	Chile	Rajchenberg et al. (2021)
*Gloeosoma mirabilis*	He3733	KY450787	KY450791	China	Dai et al. (2017b)
*Gloeosoma vitellinum*	CIEFAP 646cc	MT831039	MT831019	Argentina	Rajchenberg et al. (2021)
*Gloeosoma zealandicus*	PDD106668	MN044063	—	New Zealand	Chen et al. (2025)
*Laurilia sulcata*	Dai15889	KY172895	KY172911	China	Liu et al. (2017)
*Megalocystidium brunneum*	He3407	MW533077	MW528912	China	Xu et al. (2025)
*Megalocystidium brunneum*	He3447^*^	MW533078	MW528913	China	Xu et al. (2025)
*Megalocystidium chinense*	Dai14769	—	MW528909	China	Xu et al. (2025)
*Megalocystidium chinense*	He4655	MW533076	MW528910	China	Xu et al. (2025)
*Megalocystidium diffissum*	Spirin 4244 H	MT477147	MT477147	Russia	Spirin et al. (2021)
*Megalocystidium effusum*	He2526^*^	MH121215	MW528911	China	Unpublished
*Megalocystidium leucoxanthum*	CBS 269.54	MH857325	MH868866	France	Vu et al. (2019)
*Megalocystidium leucoxanthum*	HK9808	AF506420	AF506420	Spain	Larsson and Larsson (2003)
*Megalocystidium luridum*	CBS 270.54	MH857326	MH868867	France	Vu et al. (2019)
*Megalocystidium luridum*	KHL8635	AF506422	AF506422	Norway	Larsson and Larsson (2003)
*Metulodontia nivea*	NH 13108	AF506423	AF506423	Sweden	Larsson and Larsson (2003)
*Parapterulicium subarbusculum*	FLOR 56456	MH260026	MH260048	UK	Leal-Dutra et al. (2018)
*Peniophora incarnata*	NH10271	AF506425	AF506425	Sweden	Larsson and Larsson (2003)
*Peniophora nuda*	LZ15-07	MT859929	—	China	Unpublished
*Scytinostroma acystidiatum*	Dai 24608	OQ689127	OQ629351	China	Zhang et al. (2023)
*Scytinostroma acystidiatum*	KUC20121019-32	KJ668461	—	South Korea	Jang et al. (2016)
*Scytinostroma alutum*	CBS 762.81	MH861482	MH873221	France	Vu et al. (2019)
*Scytinostroma alutum*	CBS 763.81	MH861483	MH873222	France	Vu et al. (2019)
*Scytinostroma artocreas*	GHL-2016-Oct	MH142900	MH204691	USA	Liu et al. (2019)
*Scytinostroma bambusinum*	JXH 596	OR510628	PP660873	China	Ji et al. (2024)
*Scytinostroma bambusinum*	JXH 643	OR510627	PP660872	China	Ji et al. (2024)
*Scytinostroma beijingense*	He 7768	OQ731943	OQ729731	China	Li et al. (2023)
*Scytinostroma boidinii*	He 5138	MK625572	MK625497	China	Li et al. (2023)
*Scytinostroma boidinii*	He 6911	OQ731934	OQ729724	China	Li et al. (2023)
*Scytinostroma caudisporum*	CBS 746.86	MH862030	NG073580	Gabon	Vu et al. (2019)
*Scytinostroma crispulum*	CBS 716.86	MH862013	MH873703	France	Vu et al. (2019)
*Scytinostroma crispulum*	CBS 717.86	MH862014	MH873704	France	Vu et al. (2019)
*Scytinostroma daweishanense*	CLZhao 17926 *	OR096194	OR461462	China	Dong et al. (2024)
*Scytinostroma decidens*	CBS 714.86	MH862011	MH873701	France	Vu et al. (2019)
*Scytinostroma decidens*	CBS 715.86	MH862012	MH873702	France	Vu et al. (2019)
*Scytinostroma duriusculum*	CBS 757.81	MH861477	MH873216	France	Vu et al. (2019)
*Scytinostroma duriusculum*	CBS 758.81	MH861478	MH873217	France	Vu et al. (2019)
*Scytinostroma hemidichophyticum*	CBS 759.81	MH861479	MH873218	France	Vu et al. (2019)
*Scytinostroma hemidichophyticum*	CBS 760.81	MH861480	MH873219	France	Vu et al. (2019)
*Scytinostroma jacksonii*	CBS 239.87	MH862071	MH873759	Canada	Vu et al. (2019)
*Scytinostroma macrospermum*	Dai 24606	OQ689126	OQ629350	China	Zhang et al. (2023)
*Scytinostroma macrospermum*	M2138	LC327052	—	Japan	Ogura-Tsujita et al. (2018)
*Scytinostroma mediterraneense*	CBS 764.86	MH862045	MH873732	France	Vu et al. (2019)
*Scytinostroma mediterraneense*	CBS 765.86	MH862046	MH873733	France	Vu et al. (2019)
*Scytinostroma microspermum*	CBS 238.87	MH862070	—	France	Vu et al. (2019)
*Scytinostroma ochroleucum*	CBS 767.86	MH862048	—	France	Vu et al. (2019)
*Scytinostroma ochroleucum*	CBS 768.86	MH862049	MH873735	France	Vu et al. (2019)
*Scytinostroma phaeosarcum*	CBS:761.81	MH861481	MH873220	Cote d’Ivoire	Vu et al. (2019)
*Scytinostroma portentosum*	CBS 503.48	MH856447	AF518723	Canada	Vu et al. (2019)
*Scytinostroma pseudopraestans*	CBS 737.91	MH862322	MH873994	Netherlands	Vu et al. (2019)
*Scytinostroma pseudopraestans*	CBS 738.91	MH862323	MH873995	Netherlands	Vu et al. (2019)
*Scytinostroma quintasianum*	CBS 749.86	MH862031	MH873719	Netherlands	Vu et al. (2019)
*Scytinostroma quintasianum*	CBS 750.86	MH862032	MH873720	Netherlands	Vu et al. (2019)
*Scytinostroma renisporum*	CBS 771.86	MH862051	MH873738	Indonesia	Vu et al. (2019)
*Scytinostroma renisporum*	CBS 772.86	MH862052	MH873739	Indonesia	Vu et al. (2019)
***Scytinostroma sinense***	**CLZhao 30739**	**PZ013411**	**PZ013439**	**China**	**Present study**
***Scytinostroma sinense***	**CLZhao 30792**	**PZ013412**	**PZ013440**	**China**	**Present study**
***Scytinostroma sinense***	**CLZhao 30800**	**PZ013413**	**PZ013441**	**China**	**Present study**
***Scytinostroma sinense***	**CLZhao 30870**	**PZ013414**	**PZ013442**	**China**	**Present study**
***Scytinostroma sinense***	**CLZhao 30872**	**PZ013415**	—	**China**	**Present study**
***Scytinostroma sinense***	**CLZhao 37296**	**PZ013416**	—	**China**	**Present study**
***Scytinostroma sinense***	CLZhao 37303*	**PZ013417**	**PZ013443**	**China**	**Present study**
***Scytinostroma sinense***	**CLZhao 37663**	**PZ013418**	**PZ013444**	**China**	**Present study**
***Scytinostroma sinense***	**CLZhao 37700**	**PZ013419**	—	**China**	**Present study**
***Scytinostroma sinense***	**CLZhao 37741**	**PZ013420**	**PZ013445**	**China**	**Present study**
***Scytinostroma sinense***	**CLZhao 37747**	**PZ013421**	**PZ013446**	**China**	**Present study**
***Scytinostroma sinense***	**CLZhao 37760**	**PZ013422**	**PZ013447**	**China**	**Present study**
***Scytinostroma sinense***	**CLZhao 44050**	**PZ013423**	**PZ013448**	**China**	**Present study**
***Scytinostroma sinense***	**CLZhao 44258**	**PZ013424**	**PZ013449**	**China**	**Present study**
***Scytinostroma sinense***	**CLZhao 44715**	**PZ013425**	**PZ013450**	**China**	**Present study**
***Scytinostroma sinense***	**CLZhao 44767**	**PZ013426**	**PZ013451**	**China**	**Present study**
***Scytinostroma sinense***	**CLZhao 44796**	**PZ013427**	**PZ013452**	**China**	**Present study**
*Scytinostroma subduriusculum*	He 3590	MK625571	MK625499	China	Li et al. (2023)
*Scytinostroma subduriusculum*	He 4146	MK625570	MK625498	China	Li et al. (2023)
*Scytinostroma yunnanense*	CLZhao 10758	MT611445	—	China	Wang et al. (2020)
*Scytinostroma yunnanense*	CLZhao 10802	MT611446	—	China	Wang et al. (2020)
*Stereodiscus antarcticus*	MR11265	MT831048	—	Argentina	Rajchenberg et al. (2021)
*Stereodiscus limonisporus*	PDD16691	MF631156	MF631179	Australia	Unpublished
*Stereodiscus parmuliformis*	SPG3104	MT831051	MT831031	Chile	Rajchenberg et al. (2021)
*Stereodiscus patagonicus*	MA-Fungi 90714	MF631177	MF631193	Chile	Chen et al. (2025)
*Stereodiscus trivialis*	MR11264	MT831047	—	Argentina	Rajchenberg et al. (2021)
*Stereum complicatum*	He2234	KU559368	KU574828	USA	Dai and He (2016)
*Stereum gausapatum*	He1629	MH121178	MW263956	China	Unpublished
*Stereum hirsutum*	He3000	MH121188	MW263959	China	Unpublished
*Stereum insigne*	He1608	MW263980	MW263961	China	Unpublished
*Stereum insigne*	He3333	MW263981	MW263962	China	Unpublished
*Stereum lithocarpi*	He2704	MH121196	MW263963	China	Unpublished
*Stereum ochraceoflavum*	He3405	MW263982	MW263965	China	Unpublished
*Stereum ostrea*	He2067	KU559366	KU574826	USA	Dai and He (2016)
*Stereum rhododendri*	He4459	MW263993	MW263977	China	Xu et al. (2025)
*Stereum rhododendri*	He4483^*^	MW263992	MW263976	China	Xu et al. (2025)
*Stereum rugosum*	He5586	MW263984	MW263969	China	Unpublished
*Stereum sanguinolentum*	He2111	KU559367	KU574827	USA	Dai and He (2016)
*Stereum subtomentosum*	He4965	MW533090	MW528931	China	Xu et al. (2025)
*Stereum tongbiguanense*	CLZhao 42617	PV962798	PV962803	China	Chen et al. (2025)
*Stereum tongbiguanense*	CLZhao 42627*	PV962799	PV962804	China	Chen et al. (2025)
*Stereum tropicum*	He5968^*^	MW263990	MW263974	China	Xu et al. (2025)
*Stereum tropicum*	He6050	MW263991	MW263975	China	Xu et al. (2025)
*Vararia abortiphysa*	CBS:632.81	MH861387	MH873136	Gabon	Vu et al. (2019)
*Vararia ambigua*	CBS 634.81	MH861388	MH873137	France	Vu et al. (2019)
*Vararia amphithallica*	CBS:635.81	MH861389	MH873138	Gabon	Vu et al. (2019)
*Vararia amphithallica*	CBS:687.81	MH861431	MH873173	France	Vu et al. (2019)
*Vararia asiana*	CLZhao 25187*	OR102488	OR510680	China	Deng et al. (2026)
*Vararia aurantiaca*	CBS:641.81	MH861393	—	France	Vu et al. (2019)
*Vararia aurantiaca*	CBS:642.81	MH861394	MH873143	Gabon	Vu et al. (2019)
*Vararia bambusicola*	CLZhao 35740*	PV637442	PV637449	China	Deng et al. (2026)
*Vararia bambusicola*	CLZhao 35743	PV637443	PV637450	China	Deng et al. (2026)
*Vararia bannaensis*	CLZhao 35714*	PV441143	PV441157	China	Deng et al. (2026)
*Vararia bannaensis*	CLZhao 35720	PV441144	PV441158	China	Deng et al. (2026)
*Vararia breviphysa*	CBS:643.81	MH873144	MH873144	Gabon	Vu et al. (2019)
*Vararia breviphysa*	CBS:644.81	MH861396	MH873145	Gabon	Vu et al. (2019)
*Vararia calami*	CBS:646.81	MH861398	—	France	Vu et al. (2019)
*Vararia calami*	CBS:648.81	MH861399	—	France	Vu et al. (2019)
*Vararia callichroa*	CBS:744.91	MH874000	MH874000	France	Vu et al. (2019)
*Vararia cinnamomea*	CBS:641.84	MH873487	MH873487	Madagascar	Vu et al. (2019)
*Vararia cinnamomea*	CBS:642.84	MH873488	MH873488	Madagascar	Vu et al. (2019)
*Vararia cremea*	CBS:651.81	MH873147	MH873147	France	Vu et al. (2019)
*Vararia daweishanensis*	CLZhao 17911	OP380613	OP615103	China	Zou et al. (2022)
*Vararia daweishanensis*	CLZhao 17936	OP380614	OP380688	China	Zou et al. (2022)
***Vararia dehongensis***	**CLZhao 37601**	**PZ013430**	—	**China**	**Present study**
***Vararia dehongensis***	**CLZhao 37605**	**PZ013431**	**PZ013455**	**China**	**Present study**
***Vararia dehongensis***	**CLZhao 37632**	**PZ013428**	**PZ013453**	**China**	**Present study**
***Vararia dehongensis***	CLZhao 37729*	**PZ013429**	**PZ013454**	**China**	**Present study**
*Vararia dussii*	CBS:652.81	MH873148	MH873148	France	Vu et al. (2019)
*Vararia dussii*	CBS:655.81	MH861405	—	France	Vu et al. (2019)
*Vararia ellipsospora*	HHB-19503	MW740328	—	New Zealand	Zou et al. (2022)
*Vararia ferruginosa*	CLZhao 30551*	PV147175	—	China	Deng et al. (2026)
*Vararia ferruginosa*	CLZhao 30574	PV147176	PV185860	China	Deng et al. (2026)
*Vararia fissurata*	CLZhao 5218	OQ025218	OR539502	China	Deng et al. (2024b)
*Vararia fissurata*	CLZhao 8171*	OQ025219	OR539503	China	Deng et al. (2024b)
*Vararia fragilis*	CLZhao 16475	OP380612	OP380687	China	Zou et al. (2022)
*Vararia fragilis*	CLZhao 2628	OP380611	—	China	Zou et al. (2022)
*Vararia fusispora*	PDD:119539	OL709443	—	New Zealand	Zou et al. (2022)
*Vararia gallica*	CBS 234.91	MH862250	MH873932	Canada	Vu et al. (2019)
*Vararia gallica*	CBS 656.81	MH861406	MH873152	France	Vu et al. (2019)
*Vararia gillesii*	CBS:660.81	MH873153	MH873153	Netherlands	Vu et al. (2019)
*Vararia gomezii*	CBS:661.81	MH873154	MH873154	France	Vu et al. (2019)
*Vararia gracilispora*	CBS:663.81	MH861411	—	Gabon	Vu et al. (2019)
*Vararia gracilispora*	CBS:664.81	MH861412	—	Gabon	Vu et al. (2019)
*Vararia insolita*	CBS:668.81	MH861413	—	France	Vu et al. (2019)
*Vararia intricata*	CBS:673.81	MH861418	—	France	Vu et al. (2019)
*Vararia investiens*	FP 151122	MH971976	MH971977	USA	Liu and He (2018)
*Vararia investiens*	UC2023140	KP814286	—	USA	Rosenthal et al. (2017)
*Vararia isabellina*	CLZhao 22852*	OR048789	OR506350	China	Deng et al. (2024a)
*Vararia isabellina*	CLZhao 22887	OR048788	OR506351	China	Deng et al. (2024b)
*Vararia lacerata*	CLZhao 33407*	PQ811403	PV185851	China	Deng et al. (2026)
*Vararia lacerata*	CLZhao 33510	PQ811404	PV185852	China	Deng et al. (2026)
*Vararia lincangensis*	CLZhao 22791*	OR048819	OR506348	China	Deng et al. (2024b)
*Vararia lincangensis*	CLZhao 22799	OR048818	OR506349	China	Deng et al. (2024b)
*Vararia malaysiana*	CBS:644.84	MH873490	MH873490	Singapore	Vu et al. (2019)
*Vararia membaranacea*	CLZhao 35709	PV637445	PV637452	China	Deng et al. (2026)
*Vararia membaranacea*	CLZhao 35710*	PV637446	PV637453	China	Deng et al. (2026)
*Vararia minispora*	CBS:682.81	MH861426	—	France	Vu et al. (2019)
*Vararia muscicola*	CLZhao 21669*	PV147167	PV185854	China	Unpublished
*Vararia ochroleuca*	CBS:465.61	MH858109	—	France	Vu et al. (2019)
*Vararia ochroleuca*	JS24400	AF506485	AF506485	Norway	Larsson and Larsson (2003)
*Vararia parmastoi*	CBS:879.84	MH861852	MH861852	Uzbekistan	Vu et al. (2019)
*Vararia perplexa*	CBS:695.81	MH861438	—	France	Vu et al. (2019)
*Vararia pingbianensis*	CLZhao 25157	OR195737	OR510677	China	Deng et al. (2026)
*Vararia pirispora*	CBS:720.86	MH862016	—	France	Vu et al. (2019)
*Vararia punctata*	CLZhao 22439*	OR048812	OR510675	China	Deng et al. (2024b)
*Vararia rhombospora*	CBS:743.81	MH861470	—	France	Vu et al. (2019)
*Vararia rosulenta*	CBS:743.86	MH862028	—	France	Vu et al. (2019)
*Vararia rugosispora*	CBS:697.81	MH861440	—	Gabon	Vu et al. (2019)
*Vararia sigmatospora*	CBS:748.91	MH874001	MH874001	Netherlands	Vu et al. (2019)
*Vararia sinensis*	CLZhao 25160*	OR102494	OR510678	China	Deng et al. (2024b)
*Vararia sinensis*	CLZhao 25161	OR102495	OR510679	China	Deng et al. (2024b)
*Vararia sphaericospora*	CBS:700.81	MH873185	MH873185	Gabon	Vu et al. (2019)
*Vararia sphaericospora*	He4847	MK625592	MK625521	China	Unpublished
*Vararia subtropica*	CLZhao 17652	OQ025221	PX915434	China	Unpublished
*Vararia subtropica*	CLZhao 17796*	OQ025222	PX915435	China	Unpublished
*Vararia trinidadensis*	CBS:650.84	MH873495	MH873495	Madagascar	Vu et al. (2019)
*Vararia trinidadensis*	CBS:651.84	MH861803	—	Madagascar	Vu et al. (2019)
*Vararia tropica*	CBS 704.81	MH861447	MH873189	France	Vu et al. (2019)
*Vararia tuberculata*	CLZhao 41375	PX097866	PX072010	China	Wang et al. (2026)
*Vararia tuberculata*	CLZhao 41885*	PX097865	PX072017	China	Wang et al. (2026)
*Vararia vassilievae*	UC2022892	KP814203	—	USA	Unpublished
*Vararia verrucosa*	CBS:706.81	MH861449	MH861449	France	Vu et al. (2019)
*Vararia wumengshanensis*	CLZhao 31659*	PQ811405	PV185853	China	Deng et al. (2026)
*Vararia yaoshanensis*	CLZhao 20565	PP091675	PP091683	China	Deng et al. (2024b)
*Vararia yaoshanensis*	CLZhao 20693*	PP091665	PP091684	China	Deng et al. (2024b)
*Xylobolus annosus*	He3986	MH121207	MW263978	China	Cao and He (2020)
*Xylobolus austrosinensis*	He4239	MH121217	MW528945	China	Cao and He (2020)
*Xylobolus frustulatus*	He2231	MH121216	KU574825	USA	Dai and He (2016)
*Xylobolus lividocoeruleus*	FP100292	—	AY039319	USA	Wu et al. (2001)
*Xylobolus lividocoeruleus*	MB1825	—	AY039314	USA	Wu et al. (2001)
*Xylobolus princeps*	He3335	—	MW528946	China	Xu et al. (2025)
*Xylobolus subpileatus*	FP106735	—	AY039309	USA	Wu et al. (2001)
***Xylobolus yunnanensis***	CLZhao 46262*	**PZ013433**	**PZ013456**	**China**	**Present study**
***Xylobolus yunnanensis***	**CLZhao 48990**	**PZ013434**	**PZ013457**	**China**	**Present study**

The sequences were aligned in MAFFT version 7 using the G-INS-i strategy (Katoh et al. 2019). The alignment was adjusted manually using AliView version 1.27 (Larsson 2014). The dataset was initially aligned, and later, ITS and nLSU sequences were combined using Mesquite version 3.51. The sequence alignments were deposited in Figshare (doi: https://doi.org/10.6084/m9.figshare.31896226). Maximum likelihood (ML) analyses were performed using the CIPRES Science Gateway (https://www.phylo.org/portal2/login!input.action; Miller et al. 2012) based on the dataset using the RAxML-HPC BlackBox tool, with settings allowing RAxML to halt bootstrapping automatically, 0.25 for maximum hours, and the best tree obtained using ML search. Other parameters in ML analysis used default settings, and statistical support values were obtained using nonparametric bootstrapping with 1000 replicates. Bayesian inference (BI) analysis was performed on the same dataset using MrBayes v3.2.7a (Ronquist et al. 2012). The best substitution model for the dataset was selected by jModelTest2 on ACCESS v2.1.6 (Kalyaanamoorthy et al. 2017) using the Bayesian information criterion, and the model was used for Bayesian analysis. Four Markov chains were run from random starting trees. Trees were sampled every 1000^th^ generation. The first 25% of sampled trees were discarded as burn-in, while the remaining trees were used to construct a 50% majority consensus tree and to calculate Bayesian posterior probabilities (BPPs).

Phylogenetic trees were visualized and adjusted using FigTree v1.4.4 (http://tree.bio.ed.ac.uk/software/figtree), and the exports were edited using Adobe Illustrator CS6 software (Adobe Systems, USA). Branches of the consensus tree that received bootstrap support for ML equal to or above 70% and BI equal to or above 0.95 were considered well supported.

### Genealogical concordance phylogenetic species recognition (GCPSR) analysis

Genealogical concordance phylogenetic species recognition (GCPSR) analysis was employed to detect significant recombination events (Quaedvlieg et al. 2014). The data were analyzed using the pairwise homoplasy index (PHI) test in SplitsTree 4 to determine the recombination level with closely related species (Bruen et al. 2006; Huson and Bryant 2006; Quaedvlieg et al. 2014). The one-locus ITS dataset and two-locus datasets (ITS + nLSU) with closely related species were used for the analysis. A pairwise homoplasy index lower than 0.05 (Φw < 0.05) indicates significant recombination in the dataset. The relationships between closely related taxa were visualized by constructing split graphs from the concatenated datasets using the LogDet transformation and split decomposition options.

## Results

### Molecular phylogeny

**Figure 1.**
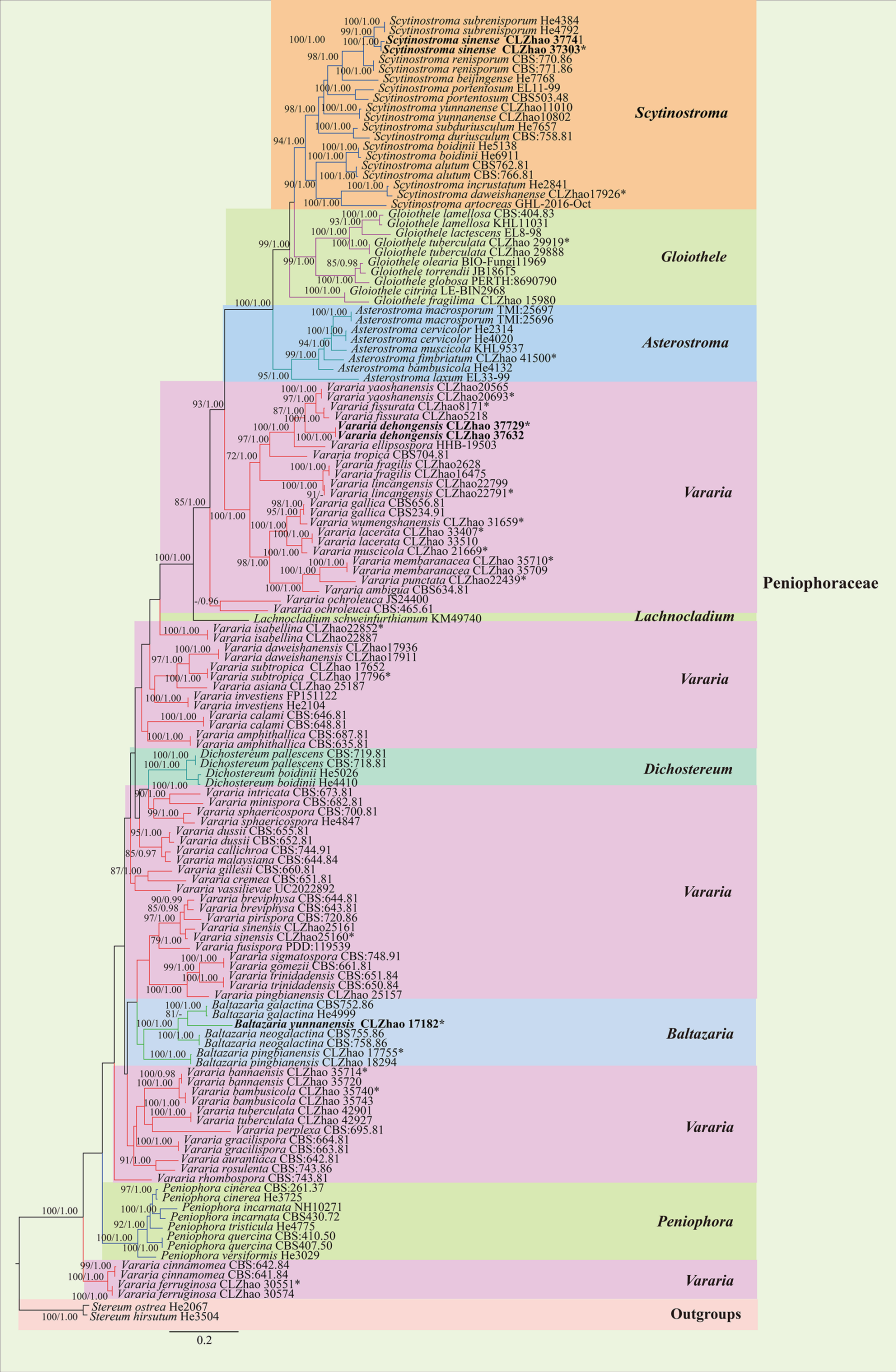
Maximum likelihood strict consensus tree illustrating the phylogeny of the family *Peniophoraceae* and related species based on ITS and nLSU sequences. Branches are labeled with ML bootstrap values higher than 70% and Bayesian posterior probabilities greater than 0.95, respectively. The new species are in bold, and type specimens are indicated with an asterisk (*).

The combined ITS + nLSU dataset (Fig. 1) comprised sequences from 135 fungal specimens representing 90 taxa, including *Asterostroma* Massee (eight specimens representing six taxa), *Baltazaria* (seven specimens representing four taxa), *Dichostereum* Pilát (four specimens representing two taxa), *Scytinostroma* (20 specimens representing 13 taxa), *Vararia* (75 specimens representing 49 taxa), *Gloiothele* Bres. (10 specimens representing eight taxa), *Lachnocladium* Lév. (one specimen representing one taxon), and *Peniophora* (eight specimens representing five taxa); *Stereum hirsutum* (Willd.) Pers. and *S. ostrea* (Blume & T. Nees) Fr. were retrieved as the outgroup taxa (Deng et al. 2024b; Deng et al. 2026). A total of four Markov chains were run for two independent runs from random starting trees, each with 3.675 million generations for the ITS + nLSU dataset, with trees and parameters sampled every 1000 generations. ML and BI analyses yielded similar topologies, with an average standard deviation of split frequencies = 0.009986 (BI) and an average effective sample size (avg. ESS) = 392.9. The phylogram based on the ITS + nLSUrDNA gene regions (Fig. 1) included eight genera that formed well-separated clades within *Peniophoraceae (Russulales)*, within which *Vararia* formed five clades, which are consistent with the results of the previous study by Deng et al. (2026). In the phylogenetic tree, two new species, *Baltazaria yunnanensis* and *Scytinostroma sinense*, were grouped into the genera *Baltazaria* and *Scytinostroma*, respectively. The species *Vararia dehongensis* was nested within the genus *Vararia*.

**Figure 2.**
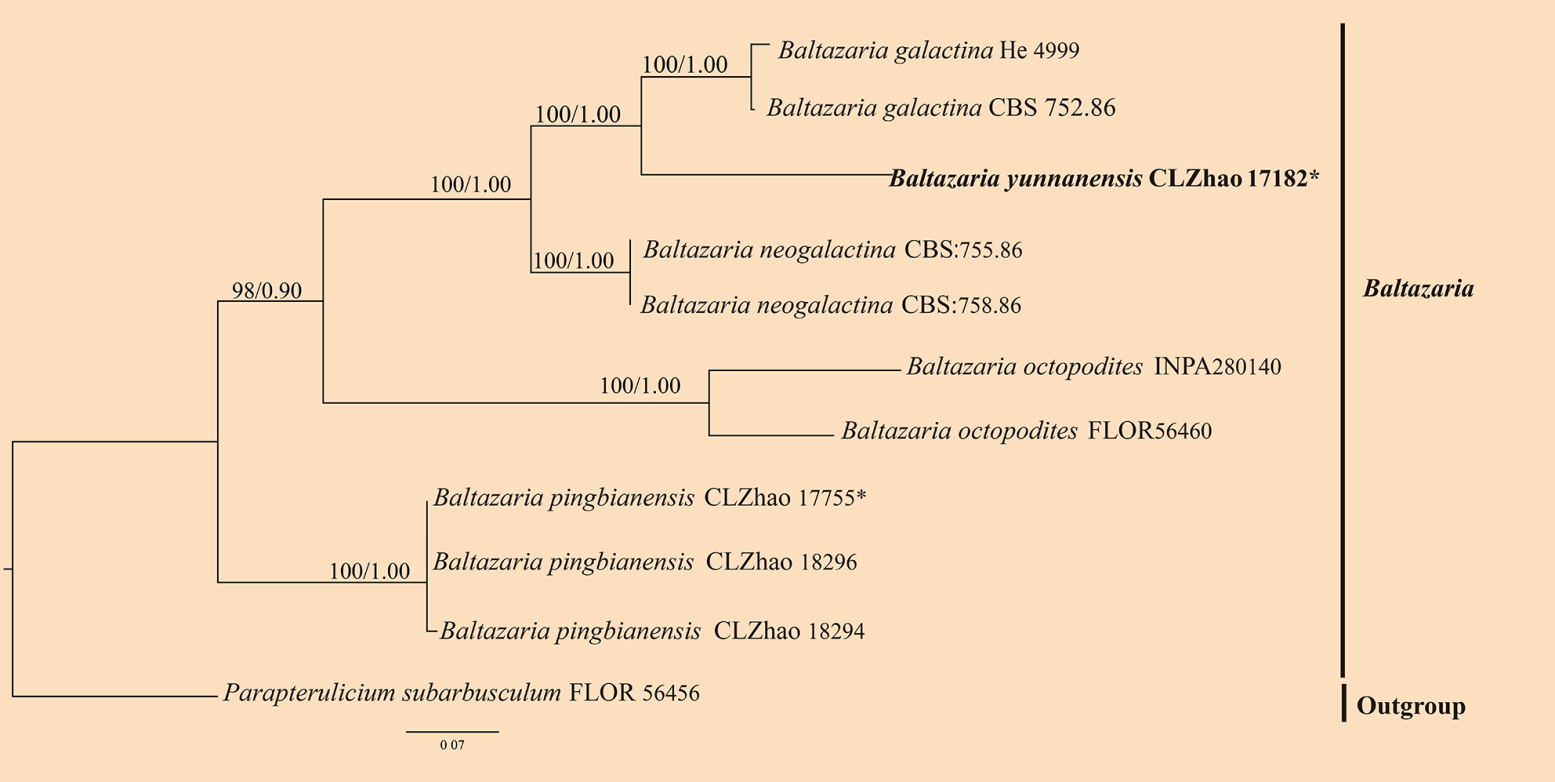
Maximum likelihood strict consensus tree illustrating the phylogeny of the genus *Baltazaria* and related species based on ITS sequences. Branches are labeled with ML bootstrap values higher than 70% and Bayesian posterior probabilities greater than 0.95, respectively. The new species are in bold, and type specimens are indicated with an asterisk (*).

For the treatment of the genus *Baltazaria*, ITS sequence data (Fig. 2) from 11 fungal specimens representing six species were included. Sequences of *Parapterulicium subarbusculum* Corner were retrieved from GenBank and used as the outgroup. A total of four Markov chains were run for two independent runs from random starting trees, each with 0.0005 million generations for the ITS data, with trees and parameters sampled every 1000 generations. JModelTest2 on ACCESS v2.1.6 (Kalyaanamoorthy et al. 2017) was used to select the best-fit model based on the BIC criterion. The best model for the ITS data estimated and applied in the Bayesian analysis was TrNef + I. ML and BI analyses yielded similar topologies, with an average standard deviation of split frequencies = 0.009304 (BI). In the ITS phylogeny (Fig. 2), the new species *Baltazaria yunnanensis* formed a distinct single lineage, closely related to *B. galactina* (Fr.) Leal-Dutra, Dentinger & G.W. Griff. and *B. neogalactina* (Boidin & Lanq.) Leal-Dutra, Dentinger & G.W. Griff.

**Figure 3.**
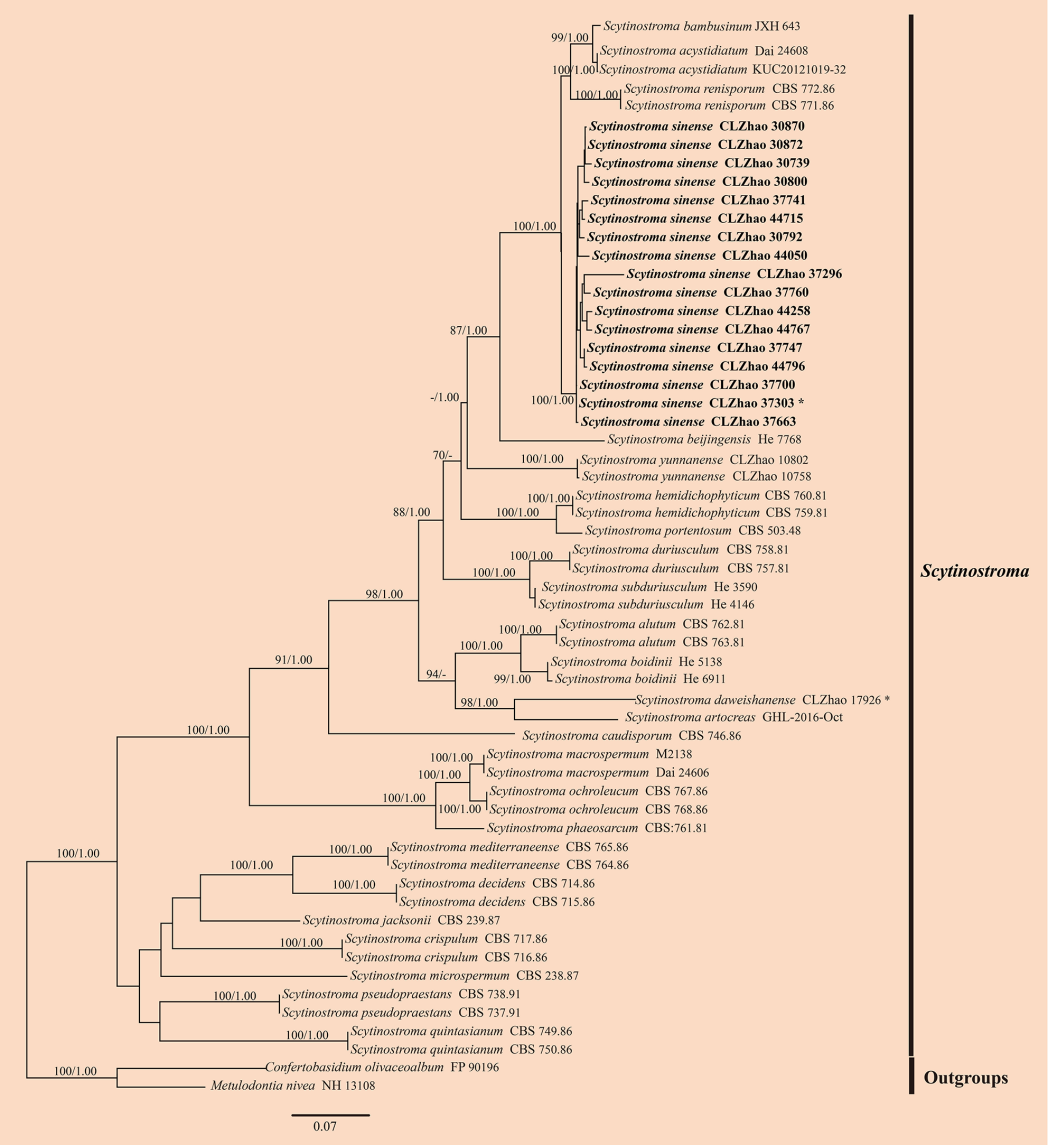
Maximum likelihood strict consensus tree illustrating the phylogeny of the genus *Scytinostroma* and related species based on ITS and nLSU sequences. Branches are labeled with ML bootstrap values higher than 70% and Bayesian posterior probabilities greater than 0.95, respectively. The new species are in bold, and type specimens are indicated with an asterisk (*).

For the analysis of the genus *Scytinostroma*, the dataset of ITS + nLSU sequences comprising sequences from 59 fungal specimens representing 27 taxa was used (Fig. 3). Sequences of *Confertobasidium olivaceoalbum* (Bourdot & Galzin) Jülich and *Metulodontia nivea* (P. Karst.) Parmasto were retrieved from GenBank and used as the outgroup (Ji et al. 2024). A total of four Markov chains were run for two independent runs from random starting trees, each with 2 million generations for the ITS + nLSU data, with trees and parameters sampled every 1000 generations. JModelTest2 on ACCESS v2.1.6 (Kalyaanamoorthy et al. 2017) was used to select the best-fit model based on the BIC criterion. The best model for the ITS + nLSU data estimated and applied in the Bayesian analysis was TIM2 + I + G. ML and BI analyses yielded similar topologies, with an average standard deviation of split frequencies = 0.009997 (BI) and an average effective sample size (avg. ESS) = 2470. The phylogenetic tree (Fig. 3) highlighted that the new species *Scytinostroma sinense* formed a distinct clade, closely related to *S. renisporum* Boidin, Lanq. & Gilles, *S. beijingense* Yue Li, S.L. Liu & S.H. He, *S. bambusinum* X.H. Ji, and *S. acystidiatum* Q.Y. Zhang, L.S. Bian & Qian Chen.

**Figure 4.**
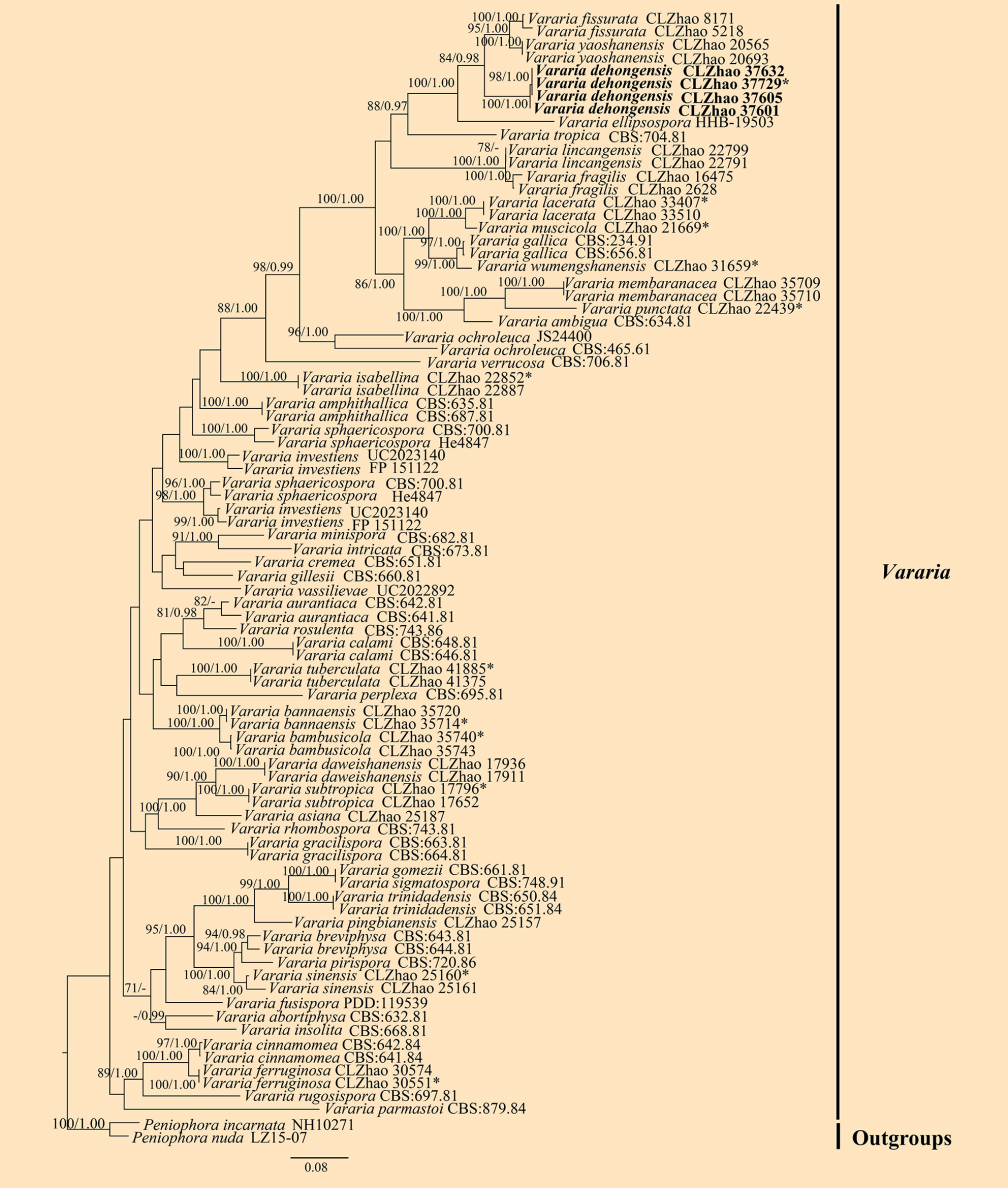
Maximum likelihood strict consensus tree illustrating the phylogeny of the genus *Vararia* and related species based on ITS and nLSU sequences. Branches are labeled with ML bootstrap values higher than 70% and Bayesian posterior probabilities greater than 0.95, respectively. The new species are in bold, and type specimens are indicated with an asterisk (*).

A combined ITS + nLSU dataset (Fig. 4) that included sequences from 86 fungal specimens representing 56 species of *Vararia* was used for the phylogenetic treatment of that genus. *Peniophora incarnata* (Pers.) P. Karst. and *P. nuda* (Fr.) Bres. were retrieved as the outgroup taxa (Deng et al. 2024b). A total of four Markov chains were run for two independent runs from random starting trees, each with 8 million generations for the ITS + nLSU dataset, with trees and parameters sampled every 1000 generations. JModelTest2 on ACCESS v2.1.6 (Kalyaanamoorthy et al. 2017) was used to select the best-fit model based on the BIC criterion. The best model for the combined ITS + nLSU dataset estimated and applied in the Bayesian analysis was TIM2 + I + G. ML and BI analyses yielded similar topologies, with an average effective sample size (avg. ESS) = 3821.6. Phylogenetic analyses based on the combined ITS + nLSU dataset (Fig. 4) revealed that the new species, *V. dehongensis*, formed a distinct clade, closely related to *V. ellipsospora* G. Cunn., *V. fissurata* Y.L. Deng & C.L. Zhao, and *V. yaoshanensis* Y.L. Deng & C.L. Zhao.

**Figure 5.**
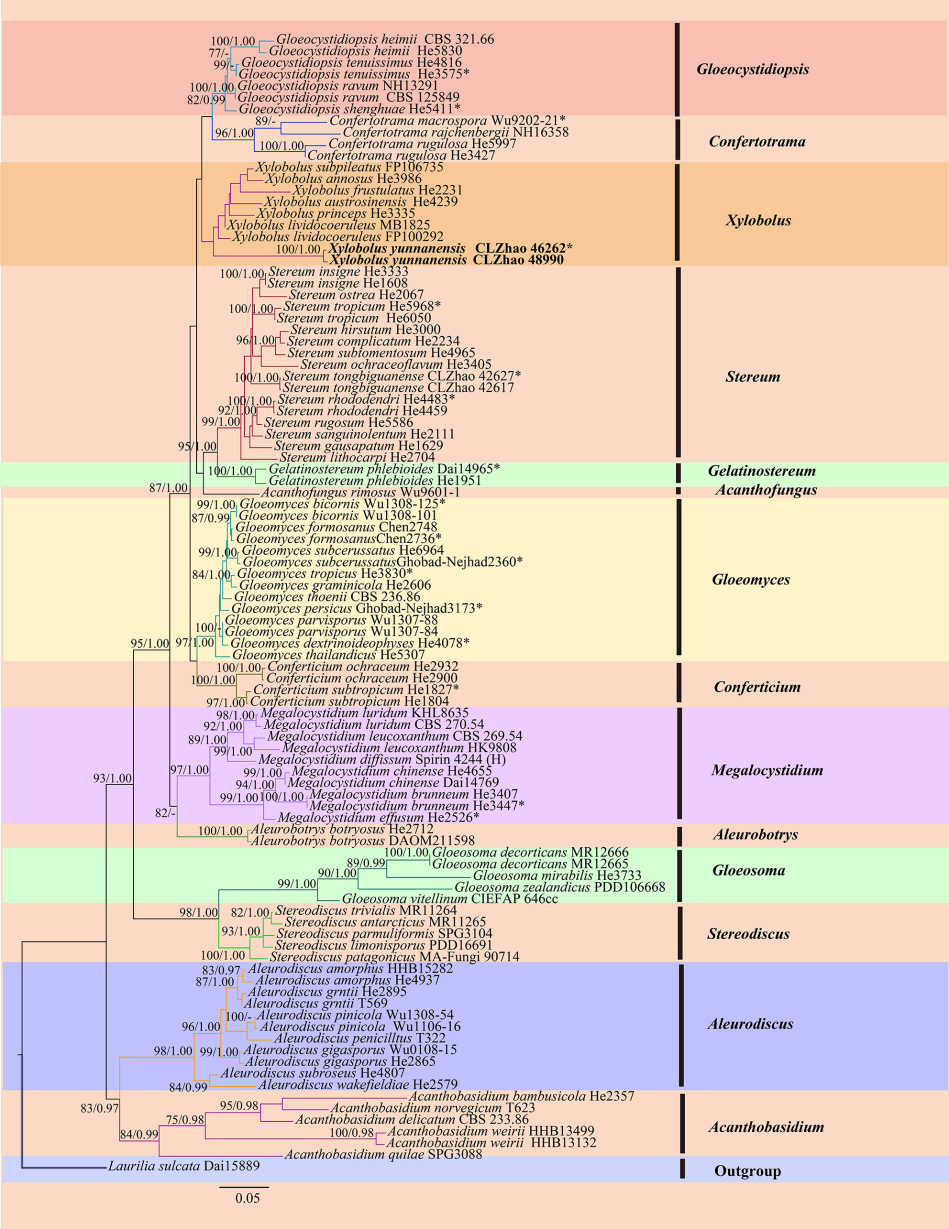
Maximum likelihood strict consensus tree illustrating the phylogeny of species of *Xylobolus* in the family *Stereaceae* and related species based on ITS and nLSU sequences. Branches are labeled with ML bootstrap values higher than 70% and Bayesian posterior probabilities greater than 0.95, respectively. The new species are in bold, and type specimens are indicated with an asterisk (*).

The combined ITS + nLSU dataset (Fig. 5) was used for the treatment of the family *Stereaceae*. It included sequences from 98 fungal specimens representing 71 species that included *Acanthobasidium* Oberw. (six specimens representing five taxa), *Acanthofungus* Sheng H. Wu, Boidin & C.Y. Chien (one specimen representing one taxon), *Aleurobotrys* Boidin (two specimens representing one taxon), *Aleurodiscus* Rabenh. ex J. Schröt. (11 specimens representing seven taxa), *Conferticium* Hallenb. (four specimens representing two taxa), *Confertotrama* Nakasone & S.H. He (four specimens representing three taxa), *Gelatinostereum* S.H. He, S.L. Liu & Y.C. Dai (two specimens representing one taxon), *Gloeocystidiopsis* Jülich (seven specimens representing four taxa), *Gloeomyces* Sheng H. Wu (14 specimens representing 10 taxa), *Gloeosoma* Bres. (five specimens representing four taxa), *Megalocystidium* Jülich (10 specimens representing six taxa), *Stereodiscus* Rajchenb. & Pildain (five specimens representing five taxa), *Stereum* (17 specimens representing 13 taxa), and *Xylobolus* (nine specimens representing seven taxa). *Laurilia sulcata* (Burt) Pouzar was retrieved as the outgroup taxon (Chen et al. 2025; Deng et al. 2026). A total of four Markov chains were run for two independent runs from random starting trees, each with 0.135 million generations for the ITS + nLSU dataset, with trees and parameters sampled every 1000 generations. JModelTest2 on ACCESS v2.1.6 (Kalyaanamoorthy et al. 2017) was used to select the best-fit model based on the BIC criterion. The best model for the combined ITS + nLSU dataset estimated and applied in the Bayesian analysis was SYM + I + G. ML and BI analyses yielded similar topologies, with an average standard deviation of split frequencies = 0.009938 (BI) and an average effective sample size (avg. ESS) = 912.5. Fourteen genera within *Stereaceae (Russulales)* formed well-separated clades in the phylogenetic tree inferred from ITS + nLSU data (Fig. 5), which is consistent with the results of the previous study by Xu et al. (2025). The phylogenetic tree showed that two specimens of the new species *Xylobolus yunnanensis* formed a distinct clade and were closely related to the genus *Xylobolus*.

**Figure 6.**
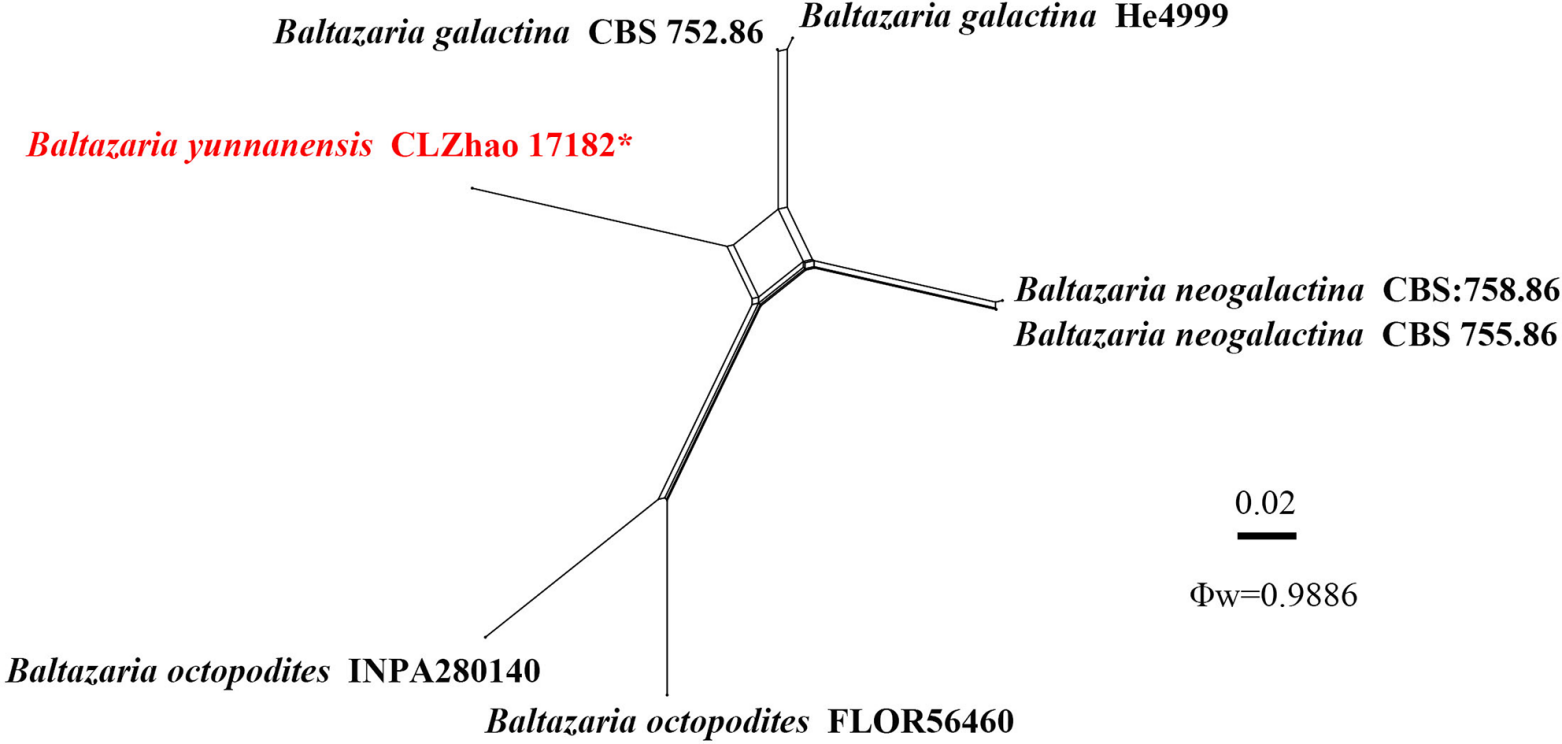
Split graphs showing the results of the PHI test for the ITS data of *Baltazaria yunnanensis* and closely related taxa using LogDet transformation and splits decomposition. PHI test results Φw ≤ 0.05 indicate significant recombination within the dataset. New taxa are in red.

**Figure 7.**
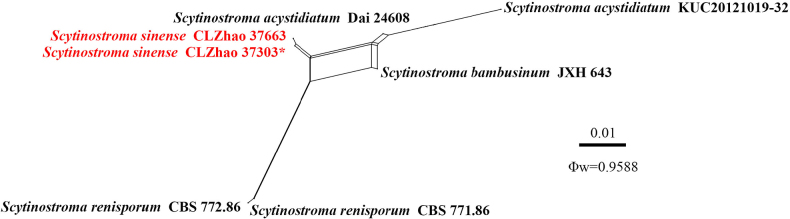
Split graphs showing the results of the PHI test for the ITS and nLSU data of *Scytinostroma sinense* and closely related taxa using LogDet transformation and splits decomposition. PHI test results Φw ≤ 0.05 indicate significant recombination within the dataset. New taxa are in red.

**Figure 8.**
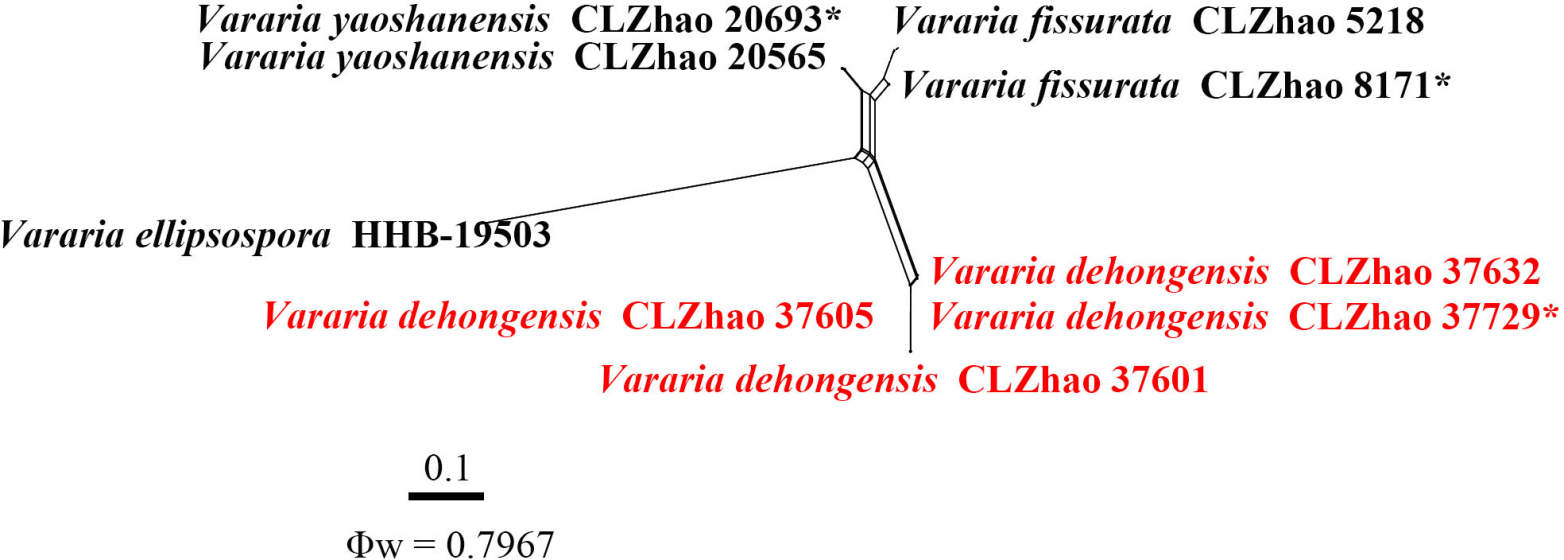
Split graphs showing the results of the PHI test for the ITS and nLSU data of *Vararia dehongensis* and closely related taxa using LogDet transformation and splits decomposition. PHI test results Φw ≤ 0.05 indicate significant recombination within the dataset. New taxa are in red.

**Figure 9.**
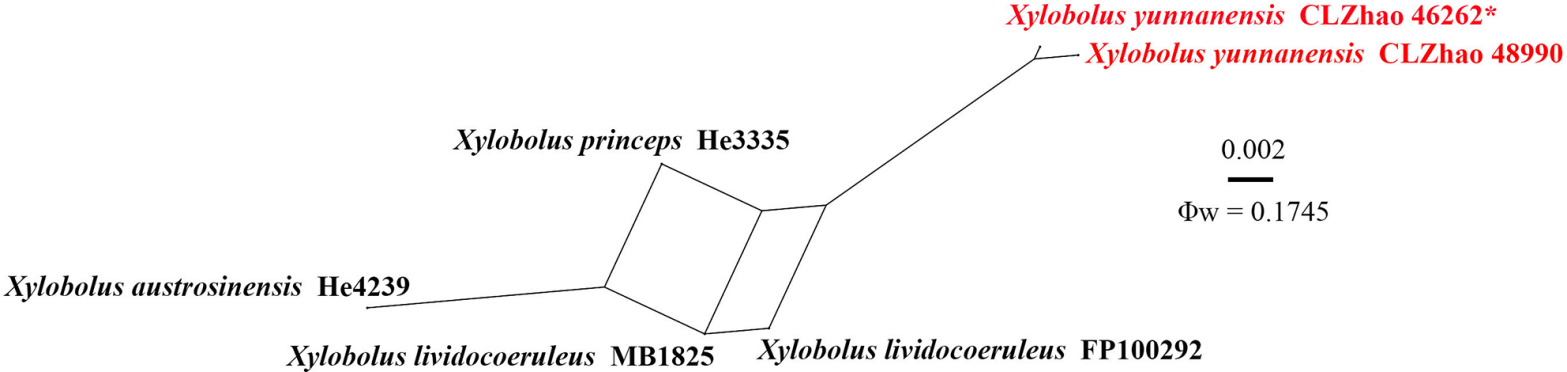
Split graphs showing the results of the PHI test for the ITS and nLSU data of *Xylobolus yunnanensis* and closely related taxa using LogDet transformation and splits decomposition. PHI test results Φw ≤ 0.05 indicate significant recombination within the dataset. New taxa are in red.

Applying the pairwise homoplasy index (PHI) test to the combined partial ITS and nLSU data tree revealed no recombination within phylogenetically related species. No significant recombination events were observed between *Baltazaria yunnanensis* and the phylogenetically closely related species *B. galactina*, *B. neogalactina*, and *B. octopodites* (Fig. 6). The test results of the combined partial ITS sequence dataset showed Φw = 0.9886 (Φw > 0.05), and no recombination was present in the new species with *B. galactina*, *B. neogalactina*, and *B. octopodites*. No significant recombination events were observed between *Scytinostroma sinense* and the phylogenetically closely related species *S. acystidiatum*, *S. bambusinum*, and *S. renisporum* (Fig. 7). The test results of the ITS + nLSU sequence dataset showed Φw = 0.9588 (Φw > 0.05), and no recombination was present in the new species with *S. acystidiatum*, *S. bambusinum*, and *S. renisporum*. No significant recombination events were observed between *Vararia dehongensis* and the phylogenetically closely related species *V. ellipsospora*, *V. fissurata*, and *V. yaoshanensis* (Fig. 8). The test results of the ITS + nLSU sequence dataset showed Φw = 0.7967 (Φw > 0.05), and no recombination was present in the new species with *V. ellipsospora*, *V. fissurata*, and *V. yaoshanensis*. No significant recombination events were observed between *Xylobolus yunnanensis* and the phylogenetically closely related species *X. austrosinensis* S.H. He, *X. lividocoeruleus*, and *X. princeps* (Jungh.) Boidin (Fig. 9). The test results of the ITS + nLSU sequence dataset showed Φw = 0.1745 (Φw > 0.05), and no recombination was present in the new species with *X. austrosinensis*, *X. lividocoeruleus*, and *X. princeps*.

### Taxonomy

#### Baltazaria yunnanensis

Taxon classification

Fungi



Y.L. Deng & C.L. Zhao sp. nov.

11F363DE-79AA-5315-B226-A67A819EE6E7

862248

Figs 10, 11, 12

##### Etymology.

*yunnanensis* (Lat.): referring to the location “Yunnan Province” where the type specimen was collected.

**Figure 10.**
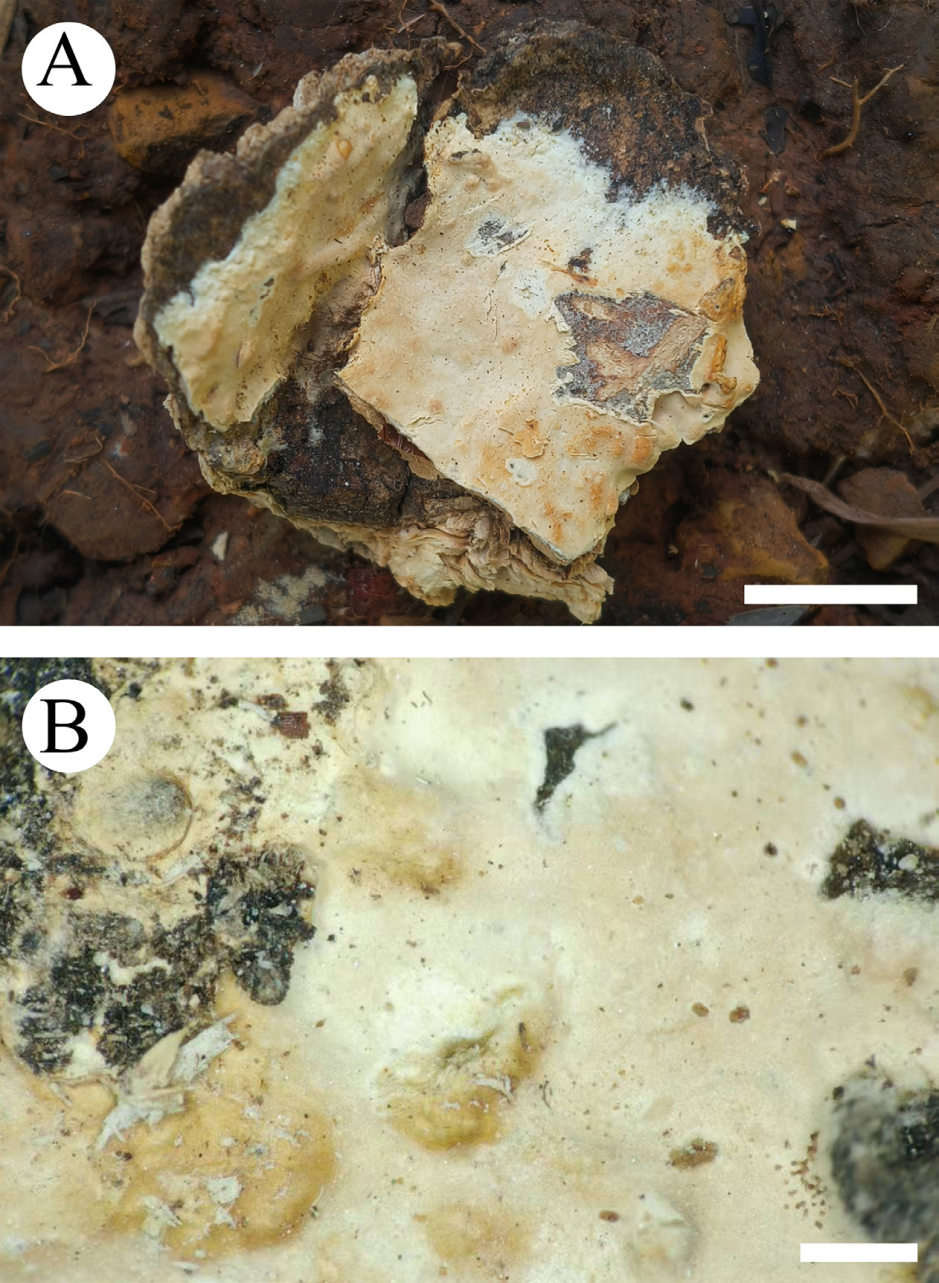
Basidiomata of *Baltazaria yunnanensis* (holotype, CLZhao 17182). **A**. Basidiomata on the substrate; **B**. Detail of the hymenophore. Scale bars: 1 cm (**A**); 1 mm (**B**).

**Figure 11.**
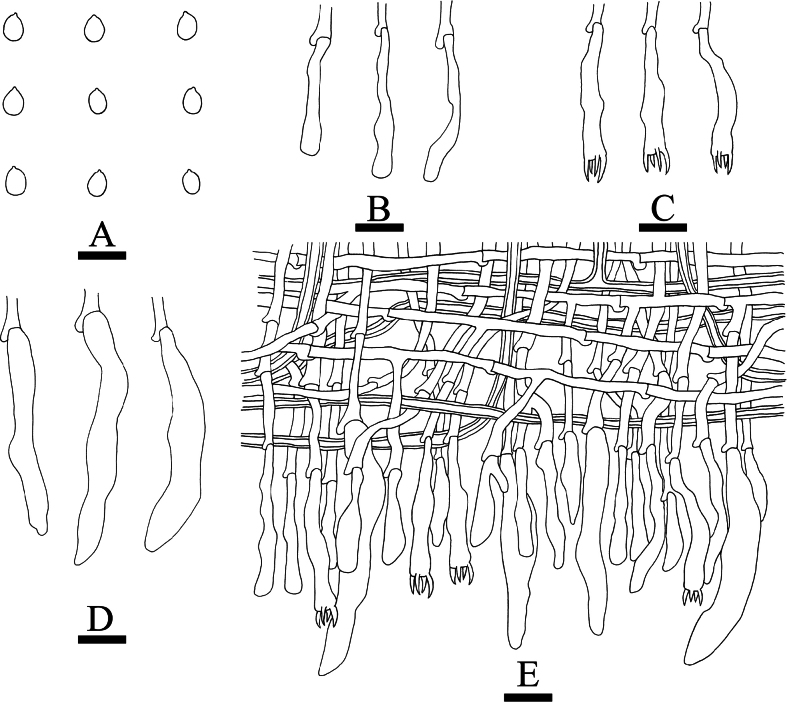
Microscopic structures of *Baltazaria yunnanensis* (holotype, CLZhao 17182). **A**. Basidiospores; **B**. Basidioles; **C**. Basidia; **D**. Cystidia; **E**. A section of the hymenium. Scale bars: 10 µm (**A–E**).

**Figure 12.**
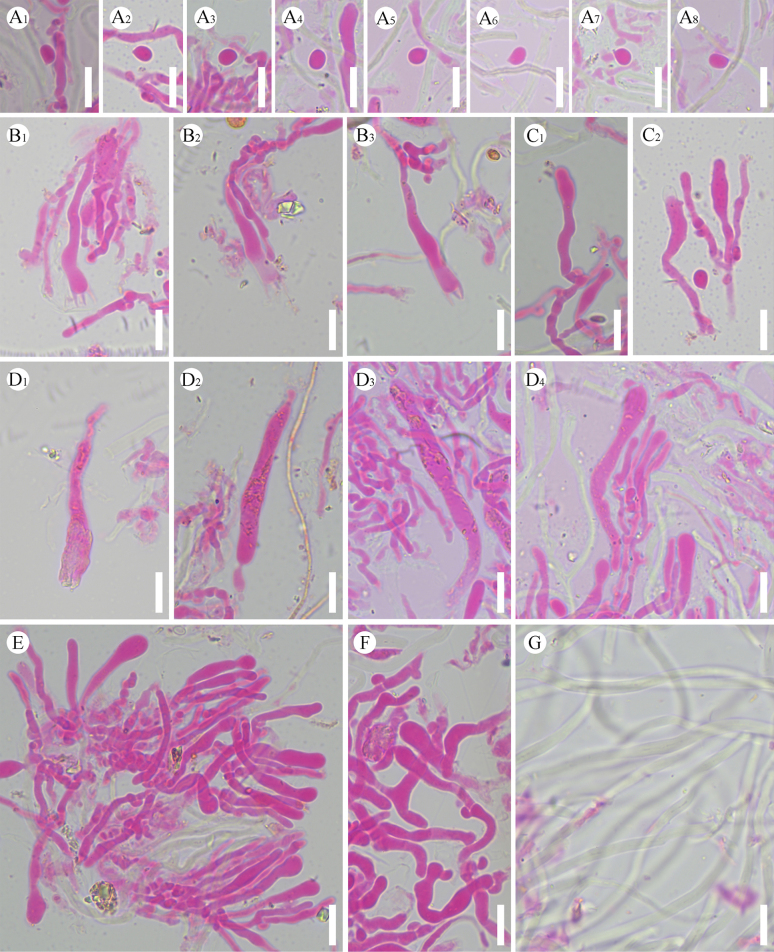
Microscopic structures of *Baltazaria yunnanensis* (holotype, CLZhao 17182). **A_1_–A_8_**. Basidiospores; **B_1_–B_3_**. Basidia; **C_1_, C_2_**. Basidioles; **D_1_–D_4_**. Cystidia; **E, F**. A section of hyphae; **G**. Skeletal hyphae. Scale bars: 10 µm (**A–G**).

##### Diagnosis.

It is characterized by its coriaceous, slightly cream to ochreous basidiomata with smooth hymenial surface, a dimitic hyphal system with clamped generative hyphae and ellipsoid to broadly ellipsoid basidiospores (4.9–5.7 × 3.8–4.7 µm).

##### Holotype.

China • Yunnan Province, Wenshan, Pingba Town, Wenshan National Nature Reserve. GPS coordinates: 23°15,0'N, 104°06'E; altitude: 1600 m asl, on angiosperm trunk, leg. C.L. Zhao, 28 July 2019, CLZhao 17182 (SWFC 00017182).

##### Basidiomata.

Annual, resupinate, coriaceous, closely adnate, without odor or taste when fresh, up to 3.5 cm long, 2.5 cm wide, and up to 100 µm thick. Hymenial surface smooth, slightly cream when fresh, cream to ochreous upon drying. Sterile margin narrow, white, up to 1 mm.

##### Hyphal system.

Dimitic, generative hyphae with clamp connections, colorless, thin-walled, smooth, interwoven, 2–2.5 µm in diameter. Skeletal hyphae colorless, smooth, 2.5–4 µm in diameter, extremely thick-walled, IKI–, CB–, tissues unchanged in KOH. Cystidia longly cylindrical, colorless, thin-walled, slightly constricted at the top, smooth, 30.7–78 × 5–9 µm. Basidia cylindrical to subclavate, with four sterigmata and a basal clamp connection, colorless, thin-walled, smooth, 26–39 × 4.5–5 µm. Basidioles dominant, similar to basidia in shape, but slightly smaller. Basidiospores ellipsoid to broadly ellipsoid, colorless, thin-walled, smooth, IKI–, CB–, (4.5–)4.9–5.7(–6.4) × (3.5–)3.8–4.7(–5) µm, *L* = 5.35 µm, *W* = 4.22 µm, *Q* = 1.27 (*n* = 60/2).

##### Material examined specimen (paratype).

China • Yunnan Province, Wenshan, Pingba Town, Wenshan National Nature Reserve. GPS coordinates: 23°15,0'N, 104°06'E; altitude: 1600 m asl, on angiosperm trunk, leg. C.L. Zhao, 28 July 2019, CLZhao 48966 (SWFC 00048966).

#### Scytinostroma sinense

Taxon classification

Fungi



Y.L. Deng & C.L. Zhao sp. nov.

BB2F7705-495D-571F-AC56-5ABA375781FF

862251

Figs 13, 14, 15

##### Etymology.

sinense (Lat.): referring to the locality (China) of the type specimen.

**Figure 13.**
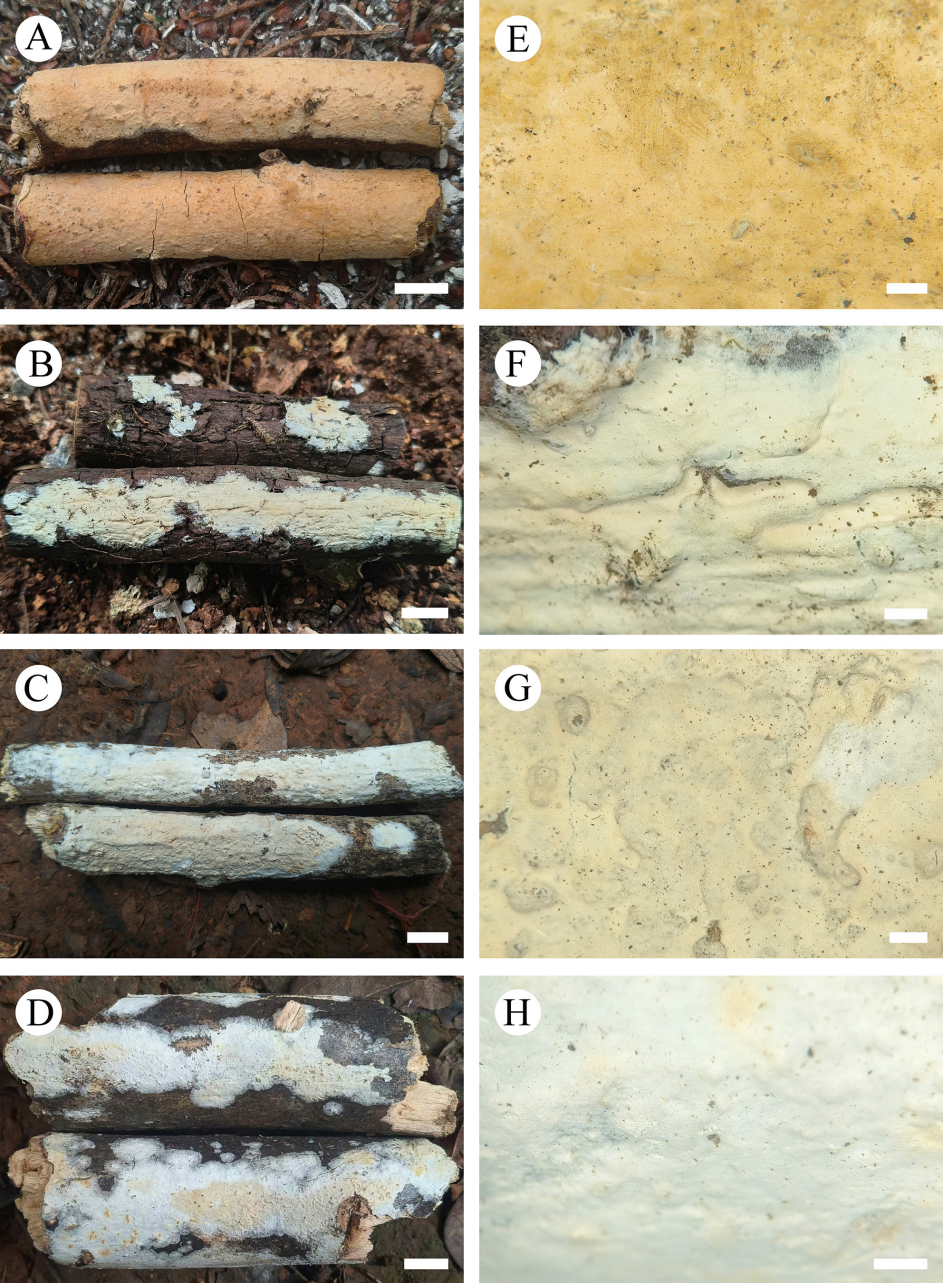
Basidiomata of *Scytinostroma sinense* (holotype, CLZhao 37303). **A–D**. Basidiomata on the substrate; **E–H**. Detail of the hymenophore; **A**, **E**. Paratype, CLZhao 30754; **B**, **F**. Paratype, CLZhao 37741; **C**, **G**. Holotype, CLZhao 37303; **D**, **H**. Paratype, CLZhao 44796. Scale bars: 1 cm (**A–D**); 1 mm (**E–H**).

**Figure 14.**
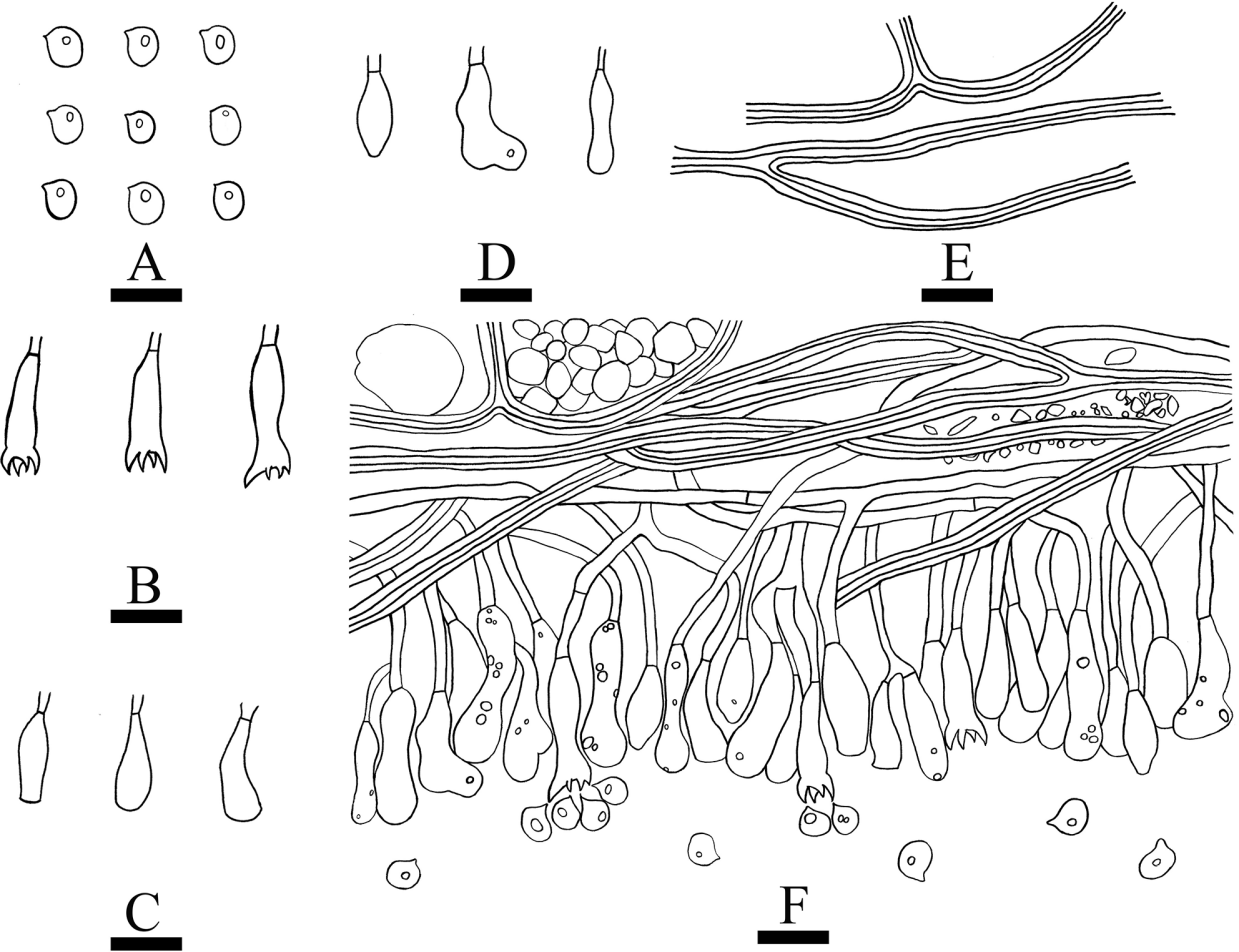
Microscopic structures of *Scytinostroma sinense* (holotype, CLZhao 37303). **A**. Basidiospores. **B**. Basidia. **C**. Basidioles. **D**. Cystidia. **E**. Skeletal hyphae. **F**. A section of the hymenium. Scale bars: 10 µm (**A–F**).

**Figure 15.**
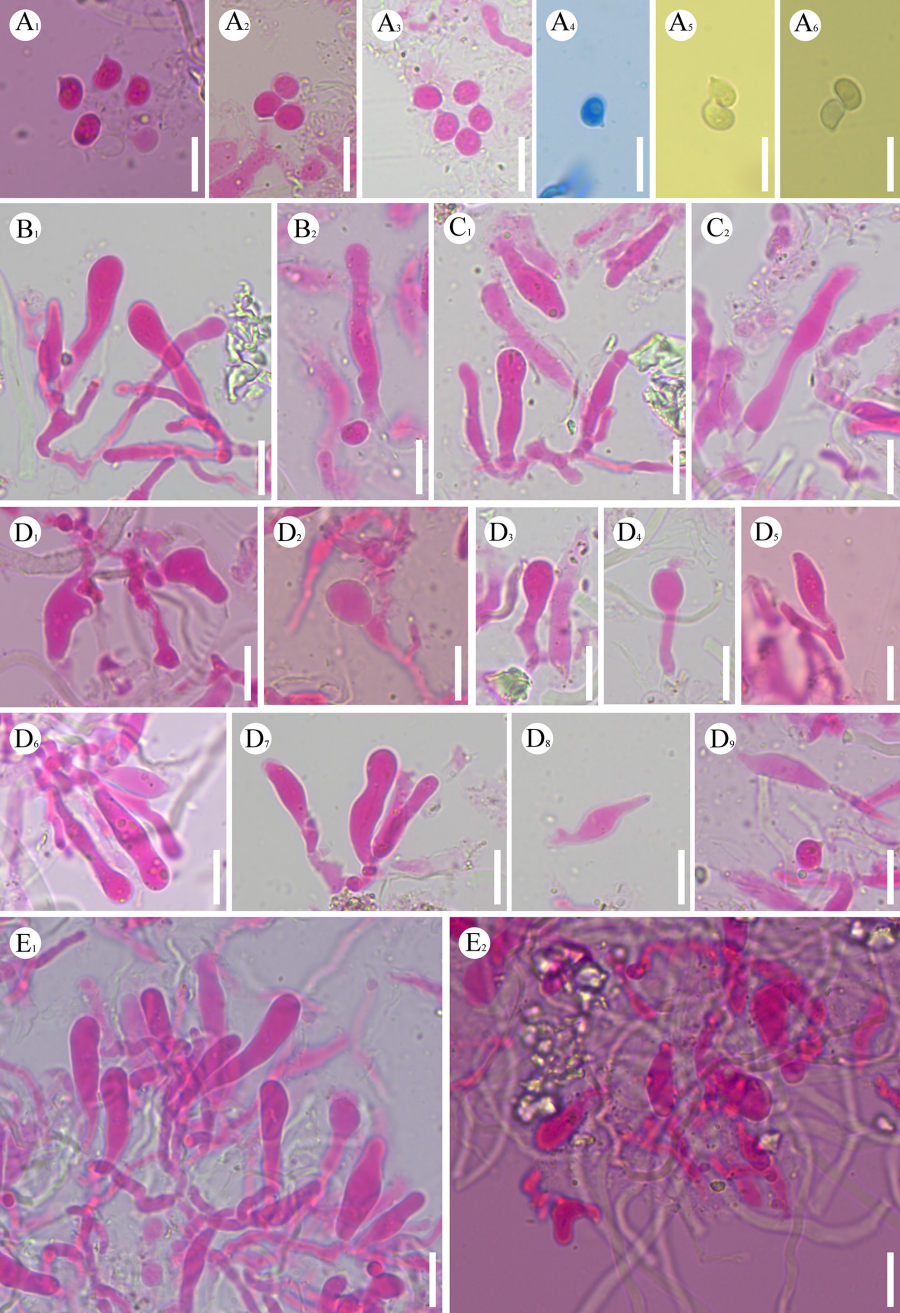
Microscopic structures of *Scytinostroma sinense* (holotype, CLZhao 37303). **A_1_–A_6_**. Basidiospores; **B_1_, B_2_**. Basidioles; **C_1_, C_2_**. Basidia; **D_1_–D_9_**. Cystidia; **E_1_, E_2_**. A section of hyphae. Scale bars: 10 µm (**A–E**).

##### Diagnosis.

*Scytinostroma sinense* is characterized by its smooth, white, cream to slightly aurantiacus hymenial surface, dimitic hyphal system with thin-walled generative hyphae bearing simple-septa and broadly ellipsoid to sublgobose basidiospores (4.9–6.1 × 3.9–5 µm).

##### Type.

China • Yunnan Province, Dehong, Yingjiang County, Tongbiguan Provincial Nature Reserve, GPS coordinates 24°71'N, 97°52'E., altitude 1500 m asl, on fallen angiosperm branch, leg. C.L. Zhao, 2 July 2024, CLZhao 37303 (SWFC 00037303).

##### Description.

Basidiomata annual, resupinate, membranous, without odor or taste when fresh, up to 9 cm long, 2.5 cm wide and 150 µm thick. Hymenial surface smooth, white to cream when fresh, cream to slightly aurantiacus upon drying. Sterile margin white, up to 1.5 mm wide.

##### Hyphal system.

Dimitic, generative hyphae bearing simple septa, thin-walled, colorless, all hyphae occasionally branched, flexuous, 1.5–4 µm in diameter, IKI–, CB–; tissues unchanged in KOH. Skeletal hyphae dominant, densely branched, smooth, distinctly thick-walled, 2–4 µm in diameter. Cystidia fusiform to capitate, colorless, smooth, thin-walled, occasionally basally inflated and slightly tapering towards the apices, 11.5–36 × 3–7 µm. Basidia clavate, colorless, thin-walled, with four sterigmata and a basal simple septum, 26–34.5 × 5–6 µm, basidioles in shape similar to basidia, but slightly smaller. Basidiospores broadly ellipsoid to sublgobose, colorless, thin-walled, smooth, IKI–, CB–, (3.6–)4.9–6.1(–7.6) × (3.2–)3.9–5(–6.1) µm, *L* = 5.49 µm, *W* = 4.4 µm, *Q* = 1.16–1.4 (*n* = 720/24).

##### Material examined specimens (paratypes).

China • Yunnan Province, Dehong, Yingjiang County, Tongbiguan Provincial Nature Reserve, GPS coordinates 24°71'N, 97°52'E., altitude 1500 m asl, on fallen angiosperm branch, leg. C.L. Zhao, 3 July 2023, CLZhao 30739 (SWFC 00030739), CLZhao 30754 (SWFC 00030754), CLZhao 30755 (SWFC 00030755), CLZhao 30769 (SWFC 00030769), CLZhao 30792 (SWFC 00030792), CLZhao 30800 (SWFC 00030800); 21 July 2023, CLZhao 30733 (SWFC 00030733), CLZhao 30736 (SWFC 00030736), CLZhao 30857 (SWFC 00030857), CLZhao 30858 (SWFC 00030858), CLZhao 30870 (SWFC 00030870), CLZhao 30872 (SWFC 00030872); 2 July 2024, CLZhao 37296 (SWFC 00037296); on the angiosperm trunk, leg. C.L. Zhao, 4 July 2024, CLZhao 37747 (SWFC 00037747), on fallen angiosperm branch, CLZhao 37760 (SWFC 00037760), CLZhao 37700 (SWFC 00037700), CLZhao 37741 (SWFC 00037741), CLZhao 37663 (SWFC 00037663). • Dehong, Ruili County, Nongdao Town, Tongbiguan Provincial Nature Reserve, GPS coordinates 23°38'N, 97°52'E, altitude 1500 m asl, on fallen angiosperm branch, leg. C.L. Zhao, 14 January 2025, CLZhao 44050 (SWFC 00044050), CLZhao 44258 (SWFC 00044258), 16 January 2025, CLZhao 44715 (SWFC 00044715), CLZhao 44767 (SWFC 00044767), CLZhao 44796 (SWFC 00044796).

#### Vararia dehongensis

Taxon classification

Fungi



Y.L. Deng & C.L. Zhao sp. nov.

D912717A-7C67-542B-AEB1-BF7C33A425D7

862252

Figs 16, 17, 18

##### Etymology.

dehongensis (Lat.): referring to the locality (Dehong) of the type specimen.

**Figure 16.**
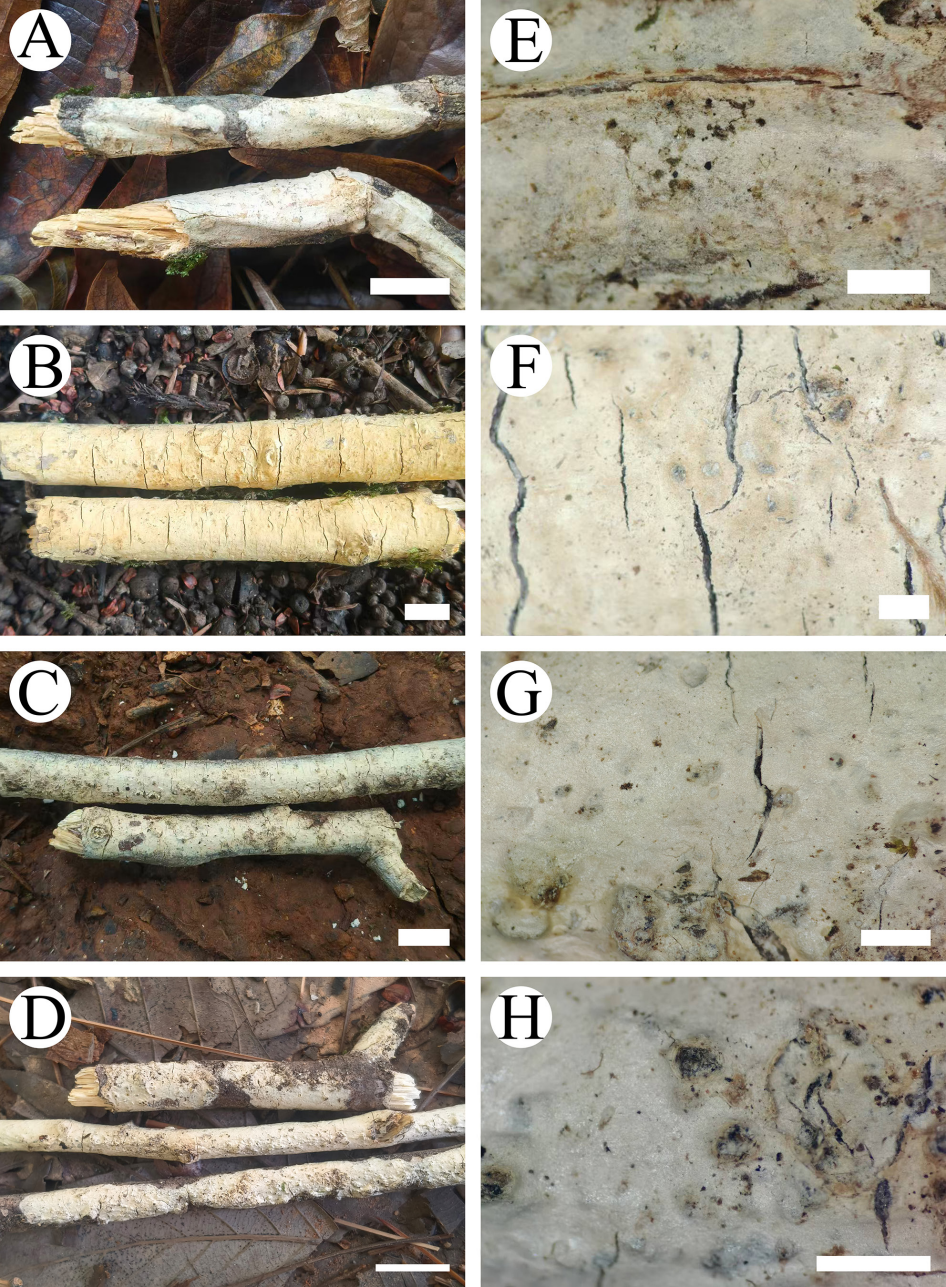
Basidiomata of *Vararia dehongensis* (holotype, CLZhao 37729). **A–D**. Basidiomata on the substrate; **E–H**. Detail of the hymenophore; **A**, **E**. Holotype, CLZhao 37729; **B**, **F**. Paratype, CLZhao 37605; **C**, **G**. Paratype, CLZhao 37632; **D**, **H**. Paratype, CLZhao 37601. Scale bars: 1 cm (**A–D**); 1 mm (**E–H**).

**Figure 17.**
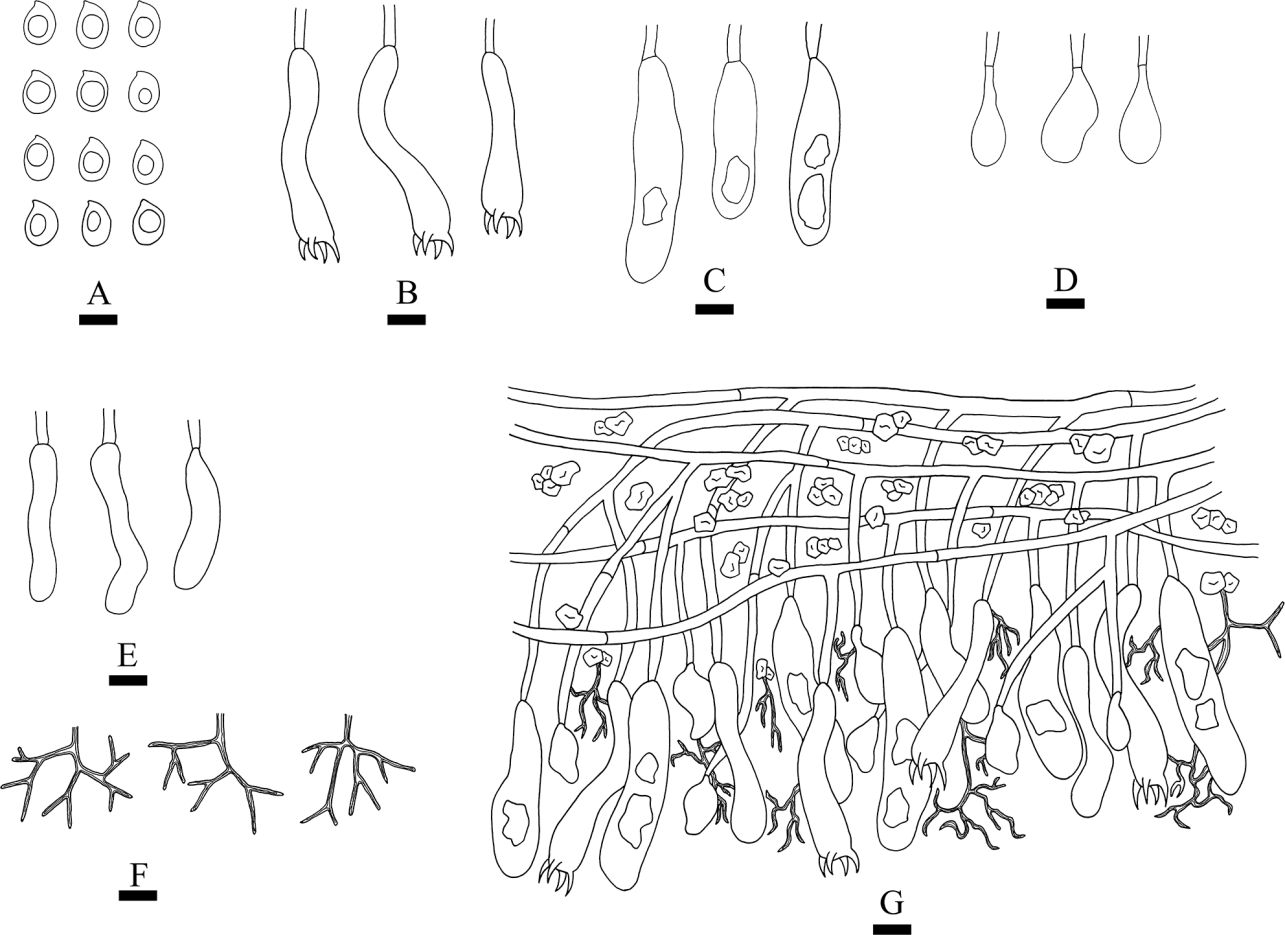
Microscopic structures of *Vararia dehongensis* (holotype, CLZhao 37729). **A**. Basidiospores; **B**. Basidia; **C**. Basidioles; **D**. Clavate gloeocystidia; **E**. Subcylindrical gloeocystidia; **F**. Dichohyphae; **G**. A section of the hymenium. Scale bars: 10 µm (**A–G**).

**Figure 18.**
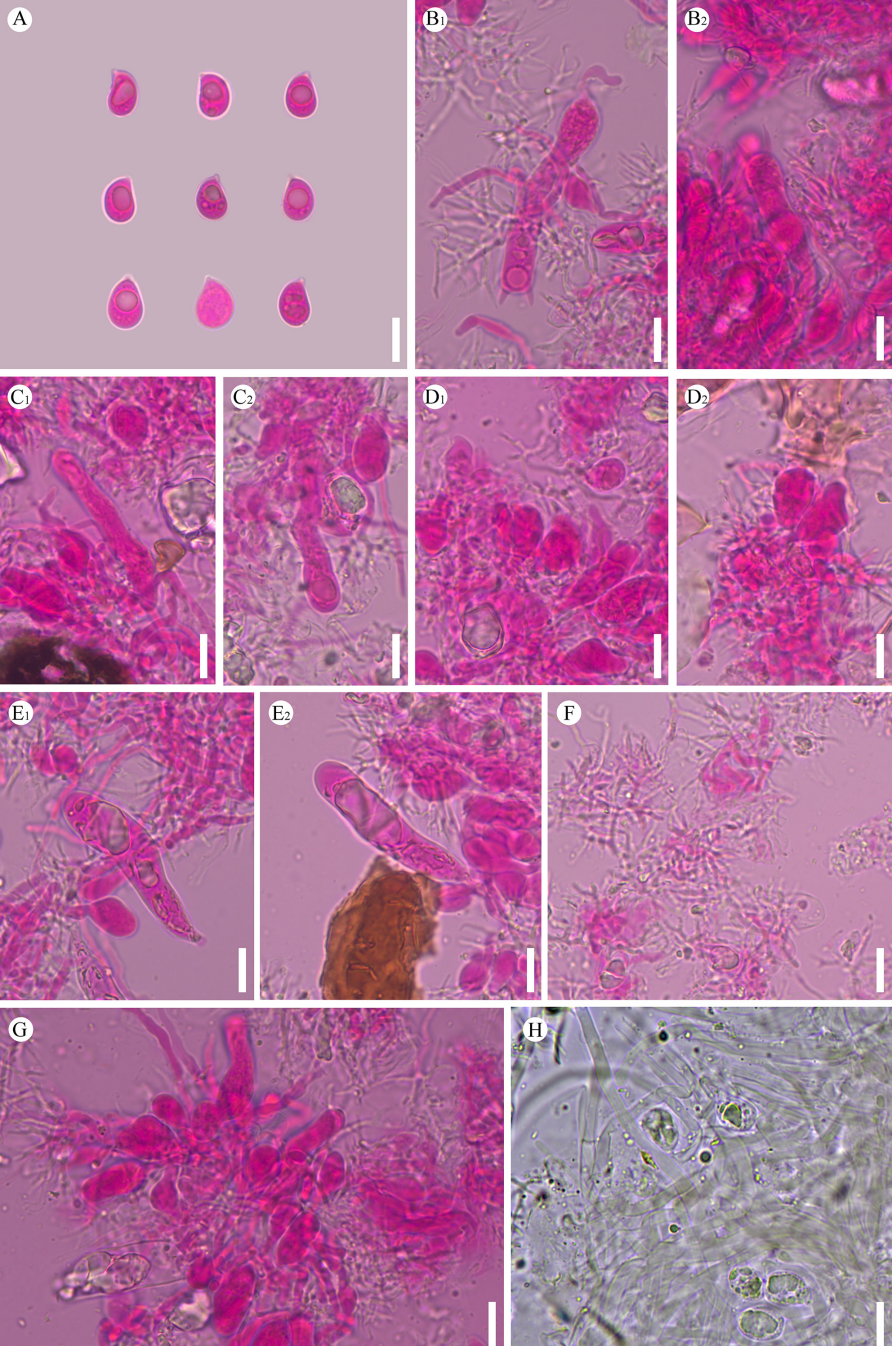
Microscopic structures of *Vararia dehongensis* (holotype, CLZhao 37729). **A**. Basidiospores; **B_1_, B_2_**. Basidia; **C_1_, C_2_**. Basidioles; **D_1_, D_2_**. Clavate gloeocystidia; **E_1_, E_2_**. Subcylindrical gloeocystidia; **F**. Dichohyphae; **G**. A section of hyphae; **H**. Generative hyphae. Scale bars: 10 µm (**A–H**).

##### Diagnosis.

*Vararia dehongensis* is characterized by the slightly cream to slightly yellowish hymenial surface, a dimitic hyphal system with generative hyphae bearing simple septa, and ellipsoid basidiospores (9–10.8 × 6–7.3 µm).

##### Type.

China • Yunnan Province, Dehong, Yingjiang County, Tongbiguan Provincial Nature Reserve, GPS coordinates 24°70'N, 97°93'E, altitude 1500 m asl, on fallen angiosperm branch, leg. C.L. Zhao, 4 July 2024, CLZhao 37729 (SWFC 00037729).

##### Description.

Basidiomata annual, resupinate, membranous, without odor or taste when fresh, up to 10 cm long, 1.5 cm wide, and up to 100 μm thick. Hymenial surface smooth, slightly cream when fresh, cream to slightly yellowish upon drying. Sterile margin white, thinning out, up to 1 mm wide.

##### Hyphal system.

Dimitic; generative hyphae bearing simple septa, colorless, thin-walled, smooth, rarely branched, interwoven, 2–3 µm in diameter, CB–; IKI–, tissues unchanged in KOH. Dichohyphae predominant, yellowish, capillary, frequently branched, thin to thick-walled, dichotomously to irregularly branched with main branches and acute tips, weakly to moderately dextrinoid in Melzer’s reagent, CB–; tissues unchanged in KOH. Gloeocystidia of two types: 1) clavate, colorless, thin-walled, smooth, 10–29.5 × 4.5–10 µm; 2) subcylindrical, colorless, thin-walled, smooth, filled with some refractive matter, 19.5–57 × 7–11 µm. Basidia subcylindrical, flexuous, with four sterigmata and a basal simple septum, colorless, thin-walled, smooth, 33–57 × 5.5–8 µm, basidioles numerous, in shape similar to basidia but smaller. Basidiospores ellipsoid, colorless, thin-walled, smooth, weak amyloid, IKI+, CB–, (7.9–)9–10.8(–11) × (5–)6–7.3(–8.4) µm, *L* = 9.87 µm, *W* = 6.72 µm, *Q* = 1.46–1.48 (*n* = 120/4).

##### Material examined specimens (paratypes).

China • Yunnan Province, Dehong, Yingjiang County, Tongbiguan Provincial Nature Reserve, GPS coordinates 24°70'N, 97°93'E, altitude 1500 m asl, on fallen angiosperm branch, leg. C.L. Zhao, 4 July 2024, CLZhao 37601 (SWFC 00037601), CLZhao 37605 (SWFC 00037605), CLZhao 37632 (SWFC 00037632).

#### Xylobolus yunnanensis

Taxon classification

Fungi



Y.L. Deng & C.L. Zhao sp. nov.

7D88749D-84FB-5C72-88F5-B949150E8258

862250

Figs 19, 20, 21

##### Etymology.

*yunnanensis* (Lat.): referring to the location “Yunnan Province” where the type specimen was collected.

**Figure 19.**
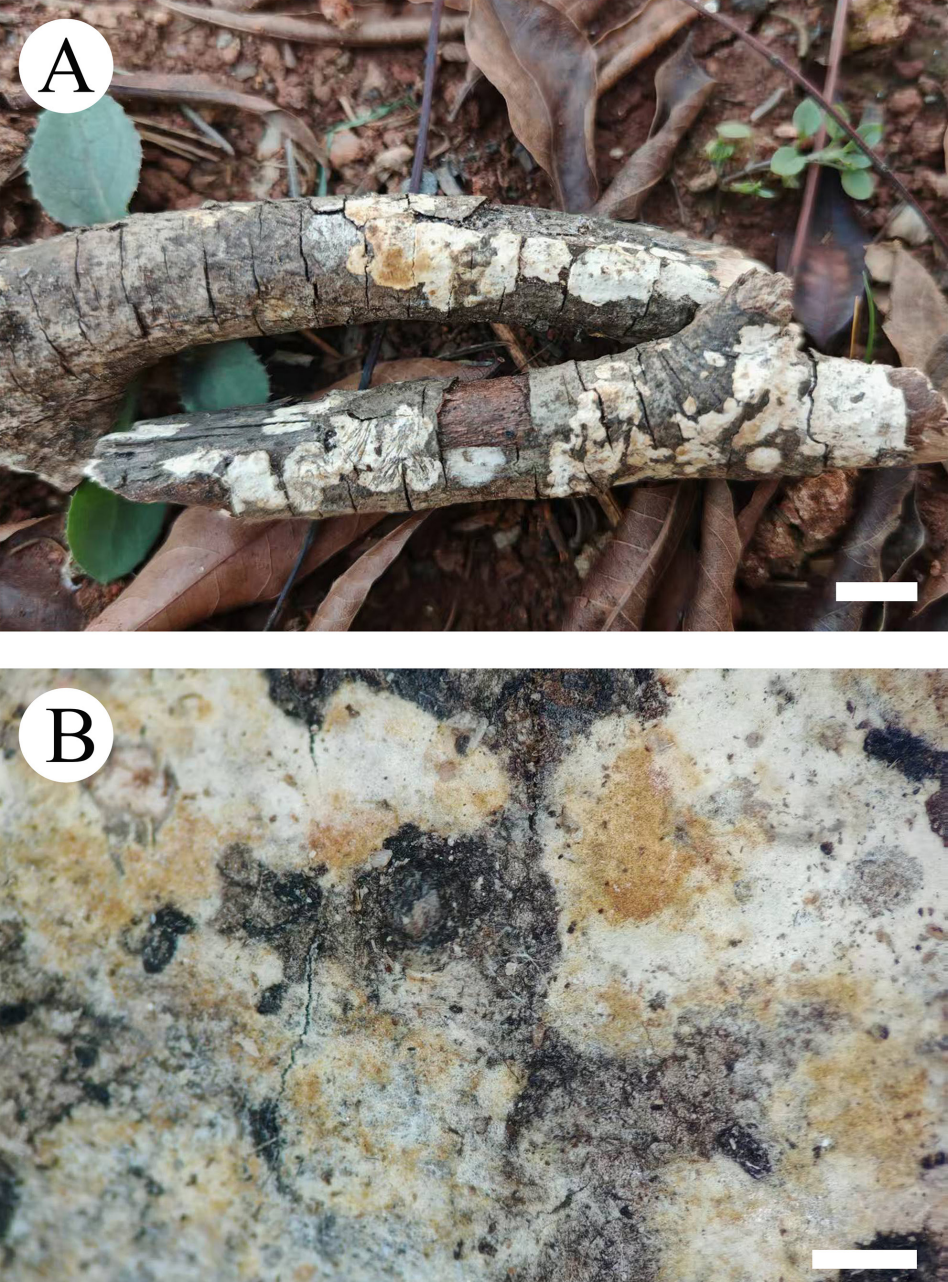
Basidiomata of *Xylobolus yunnanensis* (holotype, CLZhao 46262). **A**. Basidiomata on the substrate; **B**. Detail of the hymenophore. Scale bars: 1 cm (**A**); 1 mm (**B**).

**Figure 20.**
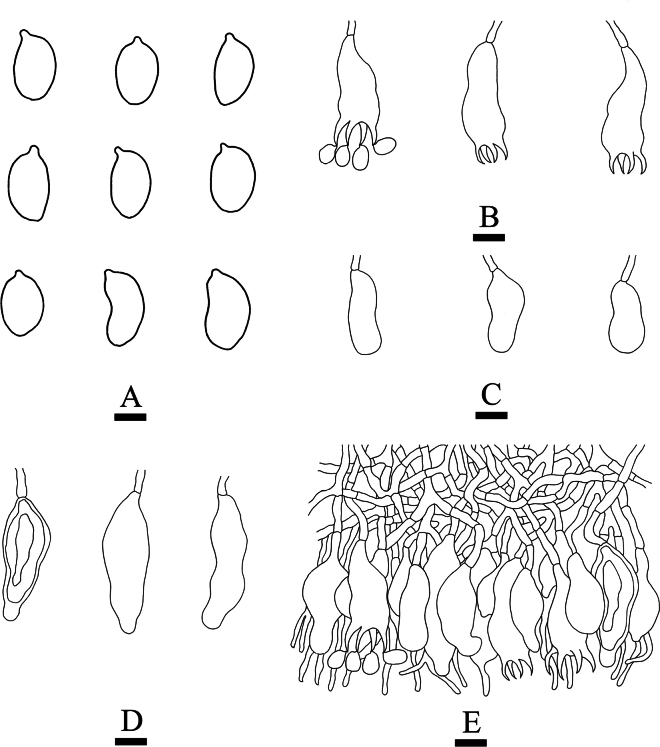
Microscopic structures of *Xylobolus yunnanensis* (holotype, CLZhao 46262). **A**. Basidiospores; **B**. Basidia; **C**. Basidioles; **D**. Gloeocystidia; **E**. A section of the hymenium. Scale bars: 5 µm (**A**); 10 µm (**B–E**).

**Figure 21.**
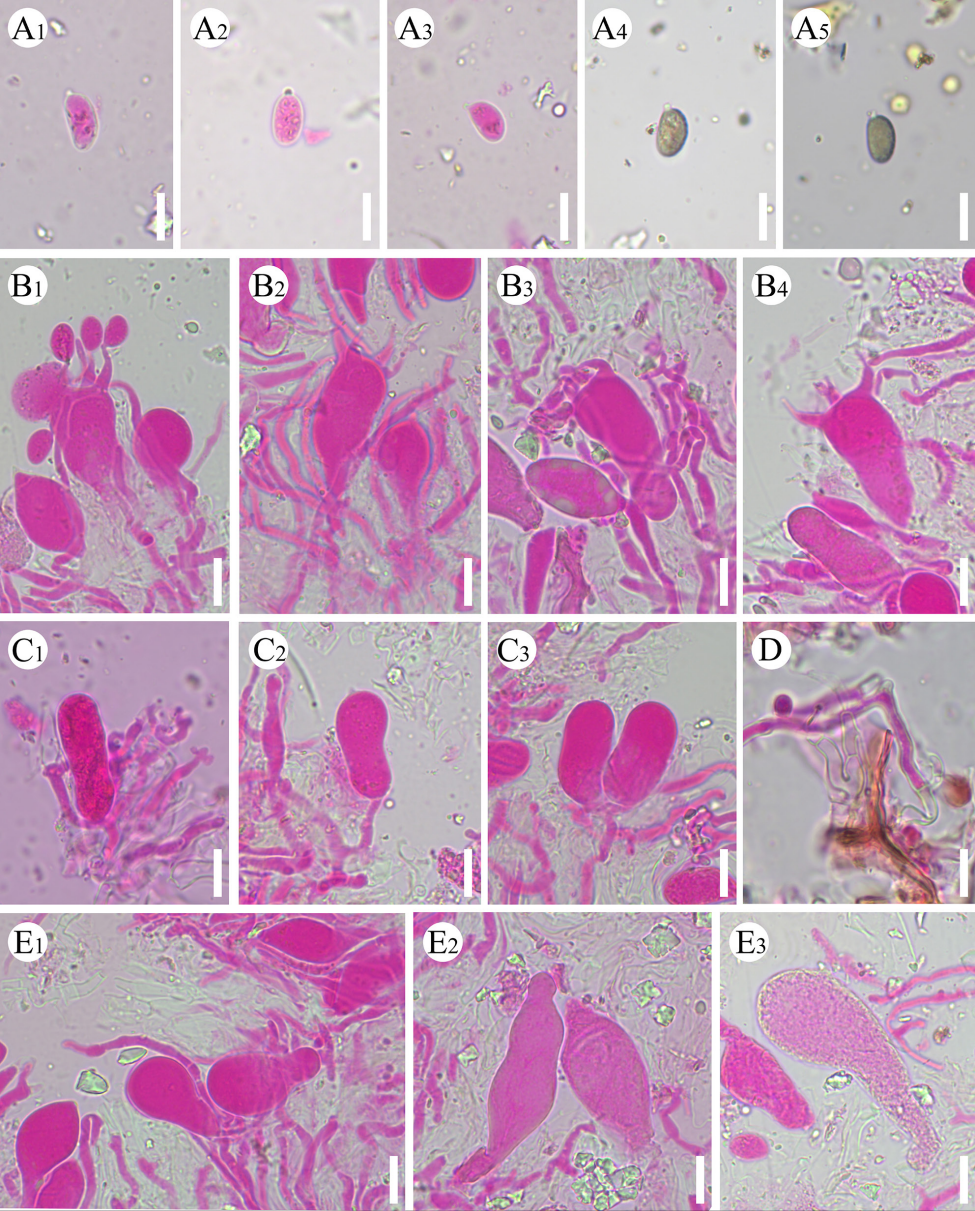
Microscopic structures of *Xylobolus yunnanensis* (holotype, CLZhao 46262). **A_1_–A_5_**. Basidiospores; **B_1_–B_4_**. Basidia; **C_1_–C_3_**. Basidioles; **D**. Generative hyphae; **E_1_–E_3_**. Gloeocystidia. Scale bars: 10 µm (**A–E**).

##### Diagnosis.

It is characterized by its membranous, slightly cream to ochreous basidiomata with smooth hymenial surface, a monomitic hyphal system with generative hyphae bearing simple septa, and basidiospores (10–12 × 6–8 µm).

##### Holotype.

China • Yunnan Province, Pu’er, Jinggu County, Mangyu Grand Canyon, GPS coordinates: 23°57'N, 100°67'E; altitude: 1600 m asl, on angiosperm branch, leg. C.L. Zhao, 20 August 2025, CLZhao 46262 (SWFC 00046262).

##### Basidiomata.

Annual, resupinate, membranous, first orbicular, later fusing together, without odor or taste when fresh, up to 10 cm long, 1.5 cm wide, and up to 100 µm thick. Hymenial surface smooth, slightly cream when fresh, cream to ochreous upon drying. Sterile margin narrow, white, up to 1 mm.

##### Hyphal system.

Monomitic; generative hyphae bearing simple septa, colorless, thin-to slightly thick-walled, smooth, interwoven, 2–3.5 µm in diameter; IKI–, CB–, tissues unchanged in KOH. Gloeocystidia numerous, variable in size and shape, urniform, fusiform, subclavate to subcylindrical, slightly constricted at the top, colorless, thin-walled, smooth, weakly dextrinoid in Melzer’s reagent, SA–, 20–69 × 11–20 µm. Basidia subcylindrical, with a basal simple septum and four sterigmata, 23–41 × 10–15 µm. Basidioles dominant, similar to basidia in shape, but slightly smaller. Basidiospores narrowly ellipsoid to allantoid, colorless, thin-walled, smooth, amyloid, IKI+, CB–, (9–)10–12(–12.7) × (5–)6–8(–8.7) µm, *L* = 11.14 µm, *W* = 6.91 µm, *Q* = 1.57–1.65 (*n* = 60/2).

##### Material examined specimen (paratype).

China • Yunnan Province, Pu’er, Jinggu County, Mangyu Grand Canyon, GPS coordinates: 23°57'N, 100°67'E; altitude: 1600 m asl, on angiosperm branch, leg. C.L. Zhao, 20 August 2025, CLZhao 48990 (SWFC 00048990).

## Discussion

Multi-locus phylogenetic analyses of *Peniophoraceae* have consistently recovered it as a well-supported monophyletic group comprising multiple distinct genera, namely *Asterostroma*, *Baltazaria*, *Dichostereum*, *Gloiothele*, *Lachnocladium*, *Michenera* Berk. & M.A. Curtis, *Peniophora*, *Scytinostroma*, *Vesiculomyces* E. Hagstr., and *Vararia*, as shown by Larsson and Larsson (2003), Larsson et al. (2004), Larsson (2007), Leal-Dutra et al. (2018), Liu and He (2018), Zou et al. (2022), Li et al. (2023), and Deng et al. (2024a, 2024b, 2026). In the combined ITS + nLSU phylogeny (Fig. 1), eight genera were represented within the family *Peniophoraceae*, and the three new species *B. yunnanensis*, *S. sinense*, and *V. dehongensis* were grouped into the genera *Baltazaria*, *Scytinostroma*, and *Vararia*, respectively. Among these, *Vararia* species were recovered in multiple clades, consistent with previous studies (Deng et al. 2024b). The ITS phylogeny of *Baltazaria* (Fig. 2) indicates that *B. yunnanensis* is sister to *B. galactina* and closely related to *B. neogalactina*. Morphologically, *B. galactina* can be delimited from *B. yunnanensis* by having both longer cylindrical gloeocystidia (30–120 × 2–5.5 µm vs. 30.7–78 × 5–9 µm) and basidia (25–50 × 3.5–4.5 µm vs. 26–39 × 4.5–5 µm) and narrower oblong to suboval basidiospores (4–5.5 × 2.8–3.5 µm vs. 4.9–5.7 × 3.8–4.7 µm) (Fries 1851; Leal-Dutra et al. 2018). *Baltazaria neogalactina* differs from *B. yunnanensis* by its beige to suede beige or even shadow beige, light tan, cinnamon basidiomata, and narrower basidiospores (4.5–6 × 3–3.5 µm vs. 4.9–5.7 × 3.8–4.7 µm) (Leal-Dutra et al. 2018). For *Scytinostroma*, the ITS + nLSU phylogeny (Fig. 3) shows that *S. sinense* formed a distinct lineage and is closely related to *S. acystidiatum*, *S. bambusinum*, *S. beijingense*, and *S. renisporum*. However, *S. acystidiatum* can be differentiated from *S. sinense* by its smooth to locally tuberculate, cream to pale yellow hymenial surface and smaller basidia (13–21 × 3.5–5 μm vs. 26–34.5 × 5–6 µm) (Zhang et al. 2023). *Scytinostroma beijingense* differs from *S. sinense* by its grayish yellow to grayish orange hymenial surface; colorless to yellow skeletal hyphae; larger thin- to slightly thick-walled ventricose (28–40 × 8–15 µm vs. 11.5–36 × 3–7 µm) and subcylindrical (45–65 × 5–7 µm vs. 11.5–36 × 3–7 µm) gloeocystidia; and wider subglobose basidiospores (4.9–6.1 × 3.9–5 µm vs. 5.5–6.5 × 5.2–6.2 µm) (Li et al. 2023). *Scytinostroma bambusinum* is distinguished from *S. sinense* by having a smooth to tuberculate, white to cream hymenial surface, shorter basidia (20–25 × 5–8 µm vs. 26–34.5 × 5–6 µm), and narrower broadly ellipsoid basidiospores (5.5–7 × 4–5.3 µm vs. 5.5–6.5 × 5.2–6.2 µm) (Ji et al. 2024). *Scytinostroma renisporum* differs from *S. sinense* by wider cylindrical, subclavate, or fusoid gloeocystidia (20–35 × 6–10 µm vs. 11.5–36 × 3–7 µm); narrower basidia (4.5–5 µm vs. 5–6 µm); and ovoid to reniform basidiospores (Leal-Dutra et al. 2018).

In *Vararia*, the phylogenetic analyses of the ITS + nLSU dataset (Fig. 4) show that the new species *V. dehongensis* is closely related to *V. ellipsospora* and *V. yaoshanensis*. Morphologically, *V. ellipsospora* differs from *V. dehongensis* by having smaller cylindrical basidia (24–30 × 5–6 µm vs. 33–57 × 5.5–8 µm) and narrower basidiospores (8–12 × 5.5–6.5 µm vs. 9–10.8 × 6–7.3 µm) (Cunningham 1955). *Vararia yaoshanensis* is distinguished from *V. dehongensis* by its cream to cinnamon-buff hymenial surface, slightly thick-walled generative hyphae, both shorter subclavate to subcylindrical basidia (23–46 × 5–8 µm vs. 33–57 × 5.5–8 µm), and slightly thick-walled basidiospores (7.6–10.8 × 5.7–7.8 µm vs. 9–10.8 × 6–7.3 µm) (Deng et al. 2024b). Based on the phylogenetic and morphological research results, 28 *Vararia* species have been reported from China, including the species newly described in the present study and those published recently (Dai 2011; Liu and He 2016; Dai et al. 2021; Zou et al. 2022; Deng and Zhao 2023; Deng et al. 2024b; Wang et al. 2026). Nevertheless, the taxonomy, phylogeny, and species boundaries of *Vararia* and allied genera remain incompletely resolved, highlighting the need for broader sampling and multilocus analyses.

Based on concatenated ITS1-5.8S-ITS2 (ITS) and D1–D2 domains of nuc 28S rDNA (LSU) sequence data, an in-depth study of the taxonomy and phylogeny of the family *Stereaceae* was conducted and revealed that 14 lineages were recognized, including the genus *Xylobolus* (Xu et al. 2025). In the present study, based on the combination of morphological features and molecular evidence, a new species, *X. yunnanensis*, is introduced within the family *Stereaceae (Russulales)*. In the phylogenetic analyses of *Stereaceae* species (Fig. 5), *X. yunnanensis* formed a distinct single lineage and is closely related to the genus *Xylobolus*. Morphologically, the new species is similar to the type species *X. frustulatus* (Pers.) P. Karst. of the genus *Xylobolus* by having a monomitic hyphal system, smooth basidia, and ellipsoid basidiospores (Bernicchia and Gorjón 2010; Ryvarden 2010, 2012; Cao and He 2020). Therefore, this taxon is tentatively assigned to *Xylobolus* pending further phylogenomic data. Morphologically, *Xylobolus yunnanensis* is similar to *X. austrosinensis* S.H. He, *X. brasiliensis* Chikowski, C.R.S. de Lira, Gibertoni & K.H. Larss., and *X. lividocoeruleus* (P. Karst.) S.H. He & Yun L. Xu by having smooth basidiospores. However, *X. austrosinensis* differs from *X. yunnanensis* by its thick-walled generative hyphae, both smaller slightly thick-walled basidia with many spines near the apex (15–20 × 2.5–4 µm vs. 23–41 × 10–15 µm) and ovoid to subglobose basidiospores (4–4.5 × 2.8–3.5 μm vs. 10–12 × 6–8 µm) (Cao and He 2020). *Xylobolus brasiliensis* can be differentiated from *X. yunnanensis* by having smaller, slightly thick-walled, yellowish to brownish, and subglobose to ellipsoid basidiospores (5–6 × 3.5–5 µm vs. 10–12 × 6–8 µm) (Crous et al. 2019). *Xylobolus lividocoeruleus* differs from *X. yunnanensis* by having thin-walled, clamped generative hyphae; both smaller subclavate basidia (20–25 × 5 µm vs. 23–41 × 10–15 µm); and subcylindrical basidiospores (7–8 × 3–4 µm vs. 10–12 × 6–8 µm) (Xu et al. 2025).

Overall, the discovery and description of the four new taxa further enrich the understanding of the species diversity of *Russulales*. Continued field surveys combined with integrative morphological and molecular approaches will undoubtedly reveal additional undescribed corticioid taxa, and future collections may also uncover species of *Peniophoraceae* and *Stereaceae* occurring on angiosperms or other host substrates.

## Supplementary Material

XML Treatment for Baltazaria yunnanensis

XML Treatment for Scytinostroma sinense

XML Treatment for Vararia dehongensis

XML Treatment for Xylobolus yunnanensis

## Additional information

### Artificial Intelligence (AI) use

The authors accept full responsibility for the content of the manuscript, including the disclosure of any use of AI.

No AI tools were used in the preparation of this manuscript.

### Author ORCIDs

Y. Deng https://orcid.org/0000-0002-8220-508X

K. Wang https://orcid.org/0009-0007-4840-816X

M. Chen https://orcid.org/0009-0005-9916-8704

Y. Qiu https://orcid.org/0009-0007-0440-903X

W. Liu https://orcid.org/0009-0008-6281-8904

S. Jabeen https://orcid.org/0000-0001-8839-7716

X. Liu https://orcid.org/0000-0003-0100-2094

C. Zhao https://orcid.org/0000-0002-8668-1075

### Data availability

All of the data that support the findings of this study are available in the main text or Supplementary Information.
